# Mutations of Human Dopamine Transporter at Tyrosine88, Aspartic Acid206, and Histidine547 Influence Basal and HIV-1 Tat-inhibited Dopamine Transport

**DOI:** 10.1007/s11481-021-09984-5

**Published:** 2021-02-03

**Authors:** Pamela M. Quizon, Yaxia Yuan, Yike Zhu, Yi Zhou, Matthew J. Strauss, Wei-Lun Sun, Chang-Guo Zhan, Jun Zhu

**Affiliations:** 1 Department of Drug Discovery and Biomedical Sciences, College of Pharmacy, University of South Carolina, 715 Sumter Street, Columbia, SC 29208, USA; 2 Molecular Modeling and Biopharmaceutical Center, University of Kentucky, Lexington, KY 40536, USA; 3 Department of Pharmaceutical Sciences, College of Pharmacy, University of Kentucky, Lexington, KY 40536, USA

**Keywords:** Dopamine transporter, HIV-1 Tat, Mutation, Uptake, Computational modeling

## Abstract

HIV-1 transactivator of transcription (Tat) has a great impact on the development of HIV-1 associated neurocognitive disorders through disrupting dopamine transmission. This study determined the mutational effects of human dopamine transporter (hDAT) on basal and Tat-induced inhibition of dopamine transport. Compared to wild-type hDAT, the maximal velocity (V_max_) of [^3^H]dopamine uptake was decreased in D381L and Y88F/D206L/H547A, increased in D206L/H547A, and unaltered in D206L. Recombinant TatR_1 – 86_ inhibited dopamine uptake in wild-type hDAT, which was attenuated in either DAT mutants (D206L, D206L/H547A, and Y88F/D206L/H547A) or mutated TatR_1 – 86_ (K19A and C22G), demonstrating perturbed Tat-DAT interaction. Mutational effects of hDAT on the transporter conformation were evidenced by attenuation of zinc-induced increased [^3^H]WIN35,428 binding in D206L/H547A and Y88F/D206A/H547A and enhanced basal MPP^+^ efflux in D206L/H547A. H547A-induced outward-open transport conformational state was further validated by enhanced accessibility to MTSET ([2-(trimethylammonium)ethyl]-methanethiosulfonate) of an inserted cysteine (I159C) on a hDAT background.. Furthermore, H547A displayed an increase in palmitoylation inhibitor-induced inhibition of dopamine uptake relative to wide-type hDAT, indicating a change in basal palmitoylation in H547A. These results demonstrate that Y88F, D206L, and H547A attenuate Tat inhibition while preserving DA uptake, providing insights into identifying targets for improving DAT-mediated dopaminergic dysregulation.

## Introduction

Despite advances in combined antiretroviral therapy, more than 50 % of HIV-1 positive individuals suffer from HIV-associated neurocognitive disorder (HAND), a spectrum of neuropsychological complications that range from mild impairment to severe dementia (Heaton et al. 2011; Clifford and Ances 2013). Because antiretroviral medications cannot efficiently cross the blood-brain barrier, chronic neuro-inflammation and neurotoxicity induced by viral proteins, such as HIV-1 trans-activator of transcription (Tat) protein that is shed from infected monocytes that enter the brain eventually lead to the development of HAND (Ensoli et al. 1993; Sulzer et al. 2005; King et al. 2006; Rayne et al. 2010). Due to the degenerative and irreversible nature of the disorder, early intervention is critical; however, there are no promising therapeutic strategies for the treatment of HAND.

Dopamine (DA) dysregulation has been implicated in the development of HAND (Purohit et al. 2011; Gaskill et al. 2017). Particularly, decreased DA transporter (DAT) expression has been observed in patients with HIV-associated dementia (Wang et al. 2004), and more so in HIV-positive patients who are concurrent abusers of cocaine (Chang et al. 2008). Our previous studies have demonstrated that *in vitro* exposure to Tat induced an inhibition of DA uptake in cells expressing hDAT (Midde et al. 2013, 2015; Quizon et al. 2016) and rat striatal synaptosomes (Zhu et al. 2009). The Tat-induced inhibition of DA uptake through human DAT (hDAT) results from a direct interaction of Tat and DAT (Midde et al. 2013; Yuan et al. 2015, 2016a). Through integrated computational modeling and experimental validation, we have identified several key residues on hDAT that are critical to the Tat-induced inhibition of DAT (Midde et al. 2013, 2015; Quizon et al. 2016). For example, single point mutation of hDAT at tyrosine88 (to phenylalanine, Y88F) preserves normal DA uptake (Midde et al. 2015), whereas DA uptake is decreased in mutation of hDAT at lysine92 (to methionine, K92M) (Midde et al. 2015) and increased in hDAT mutation at histidine547 (to alanine, H547A) (Quizon et al. 2016). Both Y88F and H547A attenuate the inhibitory effects of Tat on DAT function (Midde et al. 2015; Quizon et al. 2016). Considering that the dynamic and complex interactions between Tat and DAT involve multiple residues of DAT (Yuan et al. 2016a; Zhu et al. 2018), our recent study showed that the V_max_ values of [^3^H]DA uptake are increased in double mutant (Y88F/H547A) and decreased in triple mutant (Y88F/K92M/H547A) (Sun et al. 2019). Therefore, further determination of how Tat interacts with multiple residues on hDAT may have implications both for understanding the mechanism of Tat-regulated DAT function and developing compounds that block Tat binding to DAT. Furthermore, our previous computational modeling prediction demonstrates that either Y88 or H547 residue in hDAT could form hydrogen bond with Tat residue lysine19 or R49 on HIV-1 Tat independently (Yuan et al. 2016a). In support of the Tat-DAT interaction model, we generated mutations on the Tat protein at lysine19 residue (to alanine, K19A) to further demonstrate these key residues at Tat interacting with DAT.

Previous studies demonstrate that DAT kinetics is regulated by both phosphorylation and palmitoylation in a reciprocal manner: that is, an increase in phosphorylation or a decrease in palmitoylation reduce DA transport (Moritz et al. 2015). We have recently reported that H547A enhanced DAT uptake in a protein kinase C (PKC)-dependent manner (Quizon et al. 2016), indicating that the mutation of hDAT at His547 alters basal PKC activity. On the other hand, DA transport to the cytoplasmic pool through DAT is a dynamic conversion of the transporter conformation states (outward-open, outward-occluded, and inward-open) (Yuan et al. 2016a), which can be assessed by the accessibility of an inserted cysteine into position 159 residue (Loland et al. 2004). The accessibility of the inserted cysteine in position 159 to a compound [2-(trimethylammonium) ethyl] methanethiosulfonate (MTSET) was highly dependent upon whether the transporter was in an outward- or inward-facing conformation (Loland et al. 2003, 2004; Pedersen et al. 2014). Therefore, the addition of MTSET to a DAT construct with a mutated cysteine 159 (Ile159Cys) has been shown to result in an reduction in DA uptake, allowing the use of specific DA uptake as a functional measure for I159C reactivity (Loland et al. 2004, 2008). Based on this theory, inserting a cysteine in position 159 alongside the H547A mutation will allow the evaluation of transporter conformational states in the H547A mutant. Thus, the current study determined the palmitoylation-mediated transport capacity in H547A using an engineered cysteine in position 159 in the hDAT-H547A mutant that renders the transporter more reactive to inactivation to MTSET or 2-bromopalmitate (2-BP), a palmitoylation inhibitor.

## Materials and Methods

### Materials

[^3^H]DA (3,4-ethyl-2[N-^3^H]dihydroxyphenylethylamine; specific activity, 31 Ci/mmol), [^3^H]WIN 35,428 (specific activity, 85 Ci/mmol) and [^3^H]MPP+ (Methyl-4-Phenylpyridinium Acetate, N-[Methyl-3H]-) were purchased from PerkinElmer Life and Analytical Sciences (Boston, MA). Recombinant HIV-1 transactivator of transcription (Tat_1–86_, REP0002a) protein and its mutant Tat protein at cysteine22 residue (substituted to glycine, C22G) and at lysine19 residue (substituted to alanine, K19A) were purchased from Diatheva (Fano, Italy). Antibodies recognizing hDAT (C-20; goat polyclonal antibody), β-tubulin (H-253; rabbit monoclonal antibody) and MTSET chloride were purchased from Santa Cruz Biotechnology, Inc. (Dallas, TX). Anti-goat IgG horseradish peroxidase and anti-rabbit-IgG horseradish peroxidase were purchased from Jackson ImmunoResearch Laboratories Inc. (West Grove, PA). PC12 cells (ATCC® CRL-1721^™^), HEK293 cells (ATCC® CRL-1573^™^) and RPMI-1640 cell culture medium were purchased from American Type Culture Collection (Manassas, VA). Fetal bovine serum was purchased from Atlanta Biologicals (Flowery Branch, GA). Horse serum, penicillin/streptomycin, and trypsin/EDTA were purchased from Fisher Scientific (Waltham, MA). D-Glucose, L-ascorbic acid, cocaine, GBR 12,909, WIN 35,428, nomifensine, desipramine, DMSO, ZnCI_2_, bovine serum albumin, pyrocatechol, α-D-glucose, HEPES, isopropanol, sucrose, MTSET, 2-bromopalmitate or 2-bromohexadecanoic acid (2-BP) and Tween 20 were purchased from Sigma-Aldrich (St. Louis, MO).

### Predicting the Site for hDAT Binding With Tat

The binding structure of hDAT with HIV-1 clade B type TatR_1 – 86_ Rwas modeled and simulated based on the nuclear magnetic resonance (NMR) structures of Tat (Peloponese et al. 2000) and the previously constructed structure of the hDAT-DA complex (Midde et al. 2015; Yuan et al. 2016a). Protein docking and molecular dynamics (MD) simulation were performed to identify the conformation of hDAT-Tat complex. The energy-minimized complex structure used in this work was extracted from the long-time equilibrated MD trajectories in our previous studies (Midde et al. 2013; Yuan et al. 2015, 2016a; Quizon et al. 2016).

### Construction of Plasmids

All mutants on hDAT were generated based on the predictions made using our computational model, as described previously using site-directed mutagenesis based on the wild-type hDAT (WT hDAT) sequence (NCBI, cDNA clone MGC: 164,608 IMAGE: 40,146,999) (Quizon et al. 2016). The combination of hDAT mutations on Asp206 and His547 (Aspartic acid 206 to leucine and histidine 547 to alanine, D206L/H547A) were predicted to eliminate two hydrogen bonds between Tat and hDAT. In addition, the combination of mutations on Tyr88, Asp206, and His547 (tyrosine88 to phenylalanine and aspartic acid 206 to leucine and histidine 547 to alanine, Y88F/D206L/H547A) were predicted to eliminate three hydrogen bonds from the Tat-hDAT complex. Two extracellular cysteine90 and cysteine306 residues on WT hDAT were mutated to alanine as E2C hDAT (C90A/C306A hDAT), rendering the E2C hDAT MTSET-insensitive background (Pedersen et al. 2014). A triple mutation of the two cysteine90 and 306 residues to alanine and aspartic acid151 to cysteine on WT hDAT was generated as E2C-I159C hDAT (C90A/C306A/I159C) for MTSET-sensitive construct. A combination of C90A/C306A/H547A was generated as E2C/H547A hDAT for MTSET-insensitive control for H547A. To determine whether H547A-enhanced DA transport is mediated by changing transporter conformation, two combinations of C90A/C306A/I159C/H547A and C90A/C306A/I159A/H547A were generated as E2C-I159C/H547A and E2C-I159A/H547A for positive and negative controls for MTSET sensitive background, respectively. Synthetic cDNA encoding hDAT subcloned into pcDNA3.1+ (provided by Dr. Haley E Melikian, University of Massachusetts) was used as a template to generate mutants using site-directed mutagenesis performed by GENEWIZ (South Plainfield, NJ). DNA sequencing was also performed by GENEWIZ to confirm the sequences of the mutant constructs. Plasmid DNA were propagated and purified using the Qiagen Hi-speed maxi prep plasmid DNA isolation kit (Qiagen, Valencia, CA, USA).

### Cell Culture and Transfection

All cell cultures and transfection were conducted as described previously (Quizon et al. 2016; Sun et al. 2019). PC12 cells were maintained at 37 °C in a 5 % CO_2_ incubator in Dulbecco’s modified eagle medium (DMEM, Life Technologies, Carlsbad, CA) supplemented with 15 % horse serum, 2.5 % fetal bovine serum, 2 mM glutamine, and antibiotics (100 U/ml penicillin and 100 μg/mL streptomycin). HEK293 cells were used for the MTSET experiments and were maintained in Eagle’s minimum essential medium (MEM) supplemented with 10 % fetal bovine serum and antibiotics (100 U/ml penicillin and 100 μg/mL streptomycin). For transient transfection, cells were seeded into 24-well plates at a density of 1 × 10^5^ cells/cm^2^, or allowed to reach 100 % confluence, and were transfected 24 h later with WT hDAT or mutant hDAT plasmid DNA using Lipofectamine 2000. Cells were used for experiments 24 h after transfection.

### [^3^H]DA Uptake Assay

The maximal velocity (V_max_) and the Michaelis-Menten constant (K_m_) of [^3^H]DA uptake were examined in intact PC12 cells transiently expressing WT or mutant hDAT as previously described (Midde et al. 2013). In brief, the cells were washed twice in 1× Krebs-Ringer-HEPES (1× KRH) buffer, preincubated for 10 min at room temperature with buffer and/or 10 μM nomifensine (final concentration), and then incubated with the addition of one of six concentrations of unlabeled DA (final DA concentrations, 0.03–5 μM) and a fixed concentration of [^3^H]DA (500,000 DPM/well, specific activity, 21.2 Ci/mmol) at room temperature for 8 min. Specific uptake was calculated by subtracting nonspecific uptake (in the presence of 10 μM nomifensine and desipramine) from total uptake.

The IC_50_ of DA uptake in WT or mutant hDAT by DAT substrate and inhibitors was determined in intact PC12 cells seeded into 24-well plates as reported previously (Quizon et al. 2016). Briefly, cells were preincubated in 1× KRH buffer containing either DA (1 nM-1 mM, final concentration), GBR12909 (1 nM-10 μM, final concentration), cocaine (1 nM-1 mM, final concentration), or ZnCl_2_ (1, 10, 100 μM; final concentration) for 10 min at room temperature and then incubated for 8 min after the addition of [^3^H]DA (0.05 μM, final concentration). The cells were then washed twice with ice-cold 1× KRH buffer. Cells were lysed in 500 μl of 1 % SDS for an hour.

To determine the effects of Tat inhibition on [^3^H]DA uptake, cells were harvested and resuspended in culture medium and allowed to incubate at room temperature for 10 min. The dissociated cells were then pelleted by centrifugation at 400 × g for 5 min at 4 °C, washed once and resuspended with phosphate-buffered saline, followed by another centrifugation at 400 × g for 5 min at 4 °C, and finally resuspended in 1× KRH buffer. Specific [^3^H]DA uptake was determined in the cell suspensions prepared from WT hDAT and its mutants in the presence or absence of recombinant Tat_1 – 86_ (140 nM, final concentration). Cell suspensions were preincubated with Tat for 20 min at room temperature and then incubated for 8 min after adding [^3^H]DA (0.05 μM, final concentration). 10 μM nomifensine and desipramine (final concentration) were used to determine non-specific [^3^H]DA uptake. The incubation was terminated by immediate filtration through Whatman GF/B glass filters (presoaked with 1× KRH buffer containing 1 mM pyrocatechol for at least 3 h). Filters were washed three times with 3 ml of ice-cold KRH buffer containing pyrocatechol using a Brandel cell harvester (model M-48; Brandel Inc., Gaithersburg, MD). Radioactivity was determined as described above.

To determine whether the H547A-induced increase in V_max_ was due to a palmitoylation-mediated mechanism, kinetic analysis of [^3^H]DA uptake in WT hDAT and H547A was performed in the presence or absence of 15 μM 2-BP, a palmitoylation inhibitor. This concentration was chosen based on previous studies as well as pilot experiments conducted to determine the optimal concentration (data not shown). A 1.5 mM 2-BP stock was prepared in 100 % DMSO and was diluted to 150 μM/1 % DMSO final concentration. Intact PC12 cells in 24-well plates transiently expressing WT hDAT or H547A were washed twice with 1× KRH buffer. To determine the effects of 2-BP exposure at the zero time point (0 h), the cells were incubated for 8 min at room temperature with 15 μM 2-BP or DMSO control and six concentrations of mixed unlabeled and labeled [^3^H]DA as described above. Nonspecific uptake was determined in the presence of 10 μM nomifensine and desipramine. To dete×rmine specific uptake, nonspecific uptake was subtracted from total uptake. To determine the effects of 2-BP exposure at the 2 h time point, the cells were incubated similarly as above, but allowed to incubate at room temperature for 2 h. After each incubation, cells were washed twice with ice-cold 1× KRH buffer to terminate the r×eaction, lysed with 500 μl 1 % SDS, and allowed to shake at room temperature for an hour. Lysates were collected into scintillation vials and radioactivity was measured via liquid scintillation as above. Radioactivity was measured the next day using a liquid scintillation counter (model Tri-Carb 2900TR; PerkinElmer Life and Analytical Sciences, Waltham, MA).

### [^3^H]WIN 35,428 Binding Assay

[^3^H]WIN35,428 binding assay was conducted as described previously (Quizon et al. 2016). Binding assays were conducted to determine whether mutated hDAT alters the kinetic parameters (B_max_ or K_d_) of [^3^H]WIN 35,428 binding in intact PC12 cells transfected with WT hDAT or mutants. Cells were washed with sucrose-phosphate buffer (final concentration in mM: 2.1 NaH_2_PO_4_, 7.3 Na_2_HPO_4_7H_2_O, and 320 sucrose, pH 7.4) and then incubated with one of the six concentrations of [^3^H]WIN 35,428 (0.5–30 nM final concentrations) in a final volume of 500 μl on ice for 2 h. In parallel, nonspecific binding at each concentration of [^3^H]WIN 35,428 (in the presence of 30 μM cocaine, final concentration) was subtracted from total binding to calculate the specific binding. For the competitive inhibition experiment, assays were performed in duplicate in a final volume of 500 μl. Intact cells transfected with WT hDAT or its mutants were incubated in buffer containing 50 μl of [^3^H]WIN 35,428 (final concentration, 5 nM) and one of seven concentrations of unlabeled substrate DA (1 nM – 100 μM), cocaine (1 nM – 100 μM), GBR12909 (0.01 nM – 1 μM) or ZnCIR_2_R (1, 10, 100 μM) on ice for 2 h. Assays were terminated by removal of reaction reagents in well and then washed three times with ice-cold assay buffer. Cells were lysed with 1 % SDS for an hour. Radioactivity was determined as described above.

### Cell Surface Biotinylation and Western Blots

To determine whether the mutations alter DAT cell surface expression, biotinylation assays were performed as described previously (Zhu et al. 2005). PC12 cells transiently expressing WT hDAT or mutants were grown to 90 % confluence in 6-well plates (at a density of 1 × 10^5^ cells/cm^2^). Cells were incubated with 1 ml of 1.5 mg/ml sulfo-NHS-SS biotin (Pierce, Rockford, IL) in PBS/Ca/Mg buffer (in mM: 138 NaCl, 2.7 KCl, 1.5 KH_2_PO_4_, 9.6 Na_2_HPO_4_, 1 MgCl_2_, 0.1 CaCl_2_, pH 7.3). After incubation, cells were washed 3 times with 1 ml of ice-cold 100 mM glycine in PBS/Ca/Mg buffer and incubated for 30 min at 4 °C in 100 mM glycine in PBS/Ca/Mg buffer. Cells were then washed 3 times with 1 ml of ice-cold PBS/Ca/Mg buffer and then lysed with 500 ml of Lysis buffer (Triton X-100, 1 μg/ml aprotinin, 1 μg/ml leupeptin, 1 μM pepstatin, 250 μM phenylmethysulfonyl fluoride), followed by incubation and continuous shaking for 30 min at 4 °C. Cells were transferred to 1.5 ml tubes and centrifuged at 17,000 × g for 30 min at 4 °C. The resulting pellets were discarded, and 200 μl of the supernatants was stored at −20 °C as the total DAT fraction. The remaining supernatants were incubated with continuous shaking with ImmunoPure Immobilized Streptavidin beads in lysis buffer for 1 h at room temperature. Samples were centrifuged subsequently at 17,000 × g for 4 min at 4 °C, and supernatants (containing the non-biotinylated, intracellular protein fraction) were stored at −20 °C. Resulting pellets containing the avidin-absorbed biotinylated proteins (cell-surface fraction) were resuspended in 1 ml of 1.0 % Triton X-100 buffer and centrifuged at 17,000 × g for 4 min at 4 °C, and pellets were resuspended and centrifuged twice. Final pellets consisted of the biotinylated proteins adsorbed to monomeric avidin beads. Biotinylated proteins were eluted by incubating with 75 μl of Laemmli sample buffer for 20 min at room temperature and stored at −20 °C until western blot analysis was conducted.

To detect immunoreactive DAT protein in total, intracellular, and biotinylated fractions prepared from biotinylation assay above, samples were subjected to gel electrophoresis and Western blotting. Proteins were separated by 10 % SDS-polyacrylamide gel electrophoresis for 90 min at 150 V and transferred to Immobilon-P transfer membranes (0.45-μm pore size; Millipore, Billerica, MA) in transfer buffer (50 mM Tris, 250 mM glycine, and 3.5 mM SDS) using a Mini Trans-Blot Electrophoretic Transfer Cell (Bio-Rad Laboratories) for 110 min at 72 V. Transfer membranes were incubated with blocking buffer (5 % dry milk powder in phosphate-buffered saline containing 0.5 % Tween 20) for 1 h at room temperature, followed by incubation with primary goat anti-DAT antibody (1:200 dilution in blocking buffer) overnight at 4 °C. On next day, the transfer membranes were washed five times with wash buffer (phosphate-buffered saline containing 0.5 % Tween 20) at room temperature and then incubated with secondary anti-goat IgG horseradish peroxidase (1:3000 dilution in blocking buffer) at room temperature for 1 h. Blots on transfer membranes were detected using enhanced chemiluminescence and developed on Hyperfilm ECL-Plus (GE Healthcare, Chalfont St. Giles, Buckinghamshire, UK). After detection and quantification of DAT protein, each blot was stripped in 10 % ReBlot Plus Mild Antibody Stripping Solution (Millipore Bioscience Research Reagents, Temecula, CA) for 20 min at room temperature and reprobed for detection of β-tubulin. β-tubulin (1:2000 dilution in blocking buffer) was used as a control protein to monitor protein loading between samples and determined. Secondary anti-rabbit-IgG horseradish peroxidase (1:8000 dilution in blocking buffer) was used. Multiple autoradiographs were obtained using different exposure times, and immunoreactive bands within the linear range of detection were quantified by densitometric scanning (Scion Image software; Scion Corporation, Frederick, MD). Band density measurements, expressed as relative optical density, were used to determine levels of DAT immunoreactivity in synaptosomes.

### Basal and MPP+ Efflux Assay

Basal DA and MPP+ efflux was performed at room temperature as described previously(Midde et al. 2015). Intact PC12 cells transfected with WT hDAT or its mutants were preloaded with 0.05 μM [^3^H]DA and 0.005 μM [^3^H]MPPP^+^P for 20 min and then washed 3 times with KRH buffer prior to collecting fractional efflux samples. To obtain an estimate of the total amount of [^3^H]DA or [^3^H]MPPP^+^P in the cells at the zero time point, cells from a set of wells (four wells/sample) were lysed rapidly in 1 % SDS after preloading with [^3^H]DA or [^3^H]MPP^+^. To collect fractional efflux samples, buffer (500 μl) was added into a separate set of cell wells and transferred to scintillation vials after 1 min as an initial fractional efflux, and another 500 μl buffer was added to the same wells and collected after 10 min as second fractional efflux. Additional fractional efflux at 20, 30, 40, 50 min, respectively, was repeated under the same procedure. After the last fractional efflux, cells were lysed and counted as total amount of [^3^H]DA or [^3^H]MPP^+^ remaining in the cells from each well.

### MTSET Assay

This assay was performed based on the previous report with minor modifications (Loland et al. 2004, 2008). First, we generated five WT hDAT based plasmid construct for MTSET-insensitive or sensitive background as we described above. HEK293 cells were grown in poly-d-lysine coated plates at a density of 1 × 10^5^ cells/cm^2^ were transfected with different E2C-associated hDAT background constructs: E2C hDAT, E2C-I159C hDAT, E2C/H547A hDAT, E2C-I159C/H547A or E2C-I159A/H547A. Twenty-four hours after the transfection, cells were washed twice with 1× KRH buffer, and preincubated with or without the presence of 10 μM nomifensine (for non-specific uptake) and MTSET for 10 min at room temperature. MTSET at 1 mM final concentration was chosen based on a pilot study on optimizing MTSET concentrations (0.1, 0.5 or 1.0 mM). Previous studies show 0.5 mM MTSET for 10 min exhibiting a 40 % inhibition on E2C-I159C (Loland et al. 2004, 2008). Because MTSET is easily hydrolyzed in solution, the amount needed for a 10 × stock was weighed out and dissolved in 1 × KRH buffer just right before adding to the assigned wells, for a 10-fold dilution (final concentration of 1 mM in final volume of 250 μl). After preincubation, [^3^H]DA (0.05 μM, final concentration) was added and cells were incubated for 8 min at room temperature. Cells were immediately washed twice with ice-cold 1 × KRH buffer to terminate the reaction and were lysed rapidly with the addition of 1 % SDS. Cell lysates were collected after incubation for 1 h at room temperature and radioactivity was determined the next day.

### Data Analysis

Data are presented as means ± SEM, and *n* corresponds to the number of independent experiments performed for each group. GraphPad Prism version 5.0 was used to calculate kinetic parameters (V_max,_ K_m_, B_max_, and K_d_) from saturation curves using nonlinear regression via a Michaelis-Menten fit. IC_50_ values for substrate and inhibitors inhibiting [^3^H]DA uptake or [^3^H]WIN 35,428 binding were determined from a inhibitory curve using a one-site model with variable slope. For data involving comparisons between unpaired samples, the unpaired Student’s *t* test was used to assess differences in kinetic parameters (V_max_, K_m,_ B_max_, K_d_ or IC_50_) between WT and mutant; log-transformed values of IC_50_, K_m_ or K_d_ were used for the statistical comparisons. To determine significant differences between samples, data were analyzed with Student’s *t* tests or separate ANOVAs followed by posthoc tests, as indicated in the results and figure legends of each experiment. The kinetic parameters (V_max_, K_m,_ B_max_, K_d_ or IC_50_) were calculated using GraphPad Prism 8.0 (GraphPad Software Inc., San Diego, CA). All statistical analyses were performed using IBM SPSS Statistics version 26, with a significance threshold of *p* < 0.05.

## Results

### Computational Modeling: Mutational Effects of Asp206, Asp381, Asp206/His547 and Tyr88/Asp206/His547 on Functional Relevance of Human DAT and Tat-DAT Interaction

**Fig. 1:**
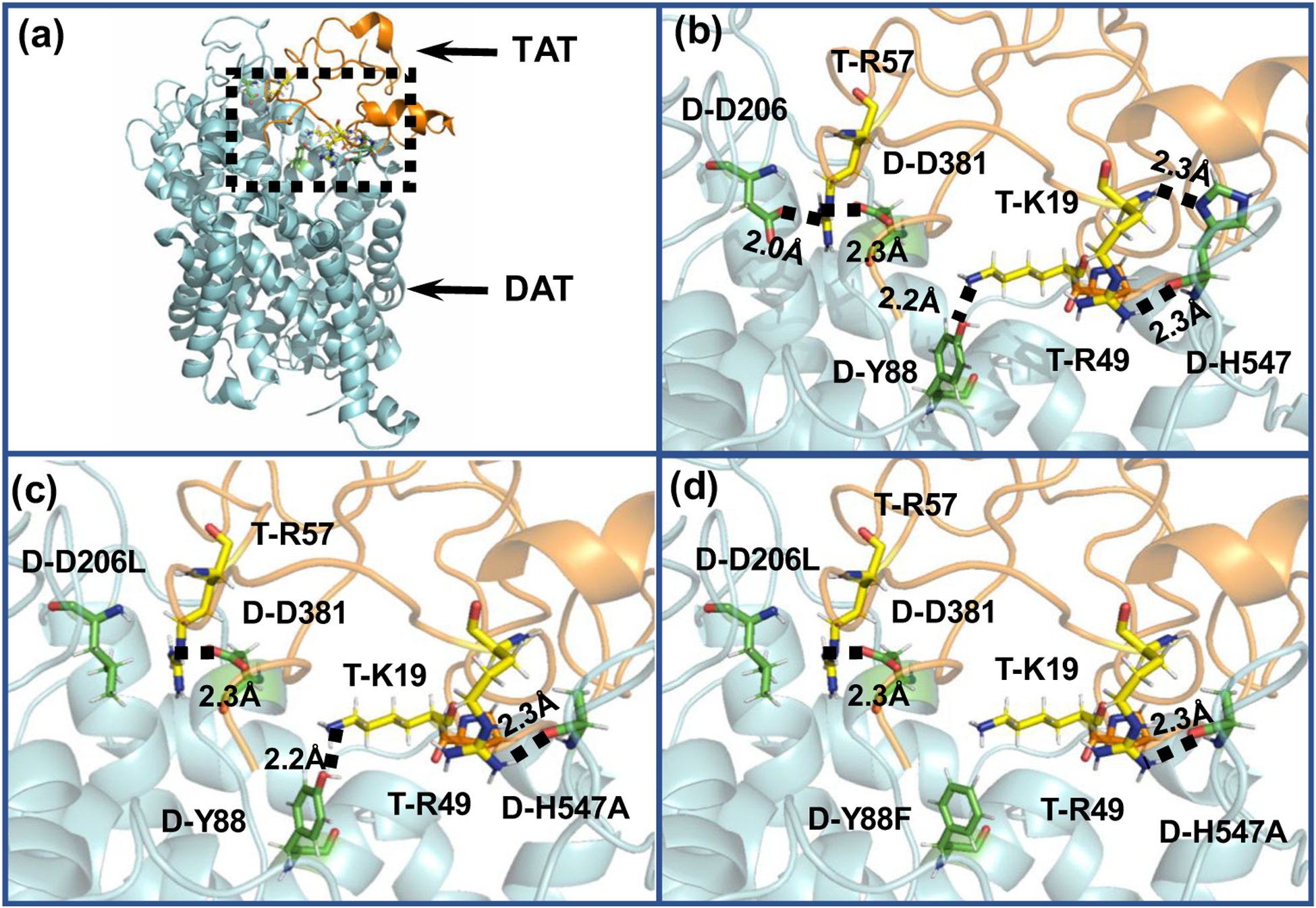
Key residues D-H547, D-Y88, and D-D206 involved in the HIV-1 Tat-DAT binding. **a** The entire complex of wild-type DAT binding with Tat from the MD trajectory. Tat and DAT are represented as gold and cyan ribbons, respectively. The dashed box indicates the binding surface between Tat and DAT. **b** The detailed interaction of Tat with wild-type DAT. Residues T-K19, T-R49, and T-R57 of HIV-1 Tat are represented in ball-stick style and colored in yellow. Residues D-H547, D-Y88, D-D206,, and D-D381 are represented in ball-stick style and colored in green. Dashed lines represent intermolecular hydrogen bonds with labeled distances; the prefix T- and D- indicate residues of Tat and DAT, respectively. **c** Tat binding with the D206L/H547A mutant of DAT. The double mutations D-D206L/D-H547A on TAT-hDAT structure. D-H547A mutation eliminates one hydrogen bond with T-R49, and D-D206L mutation eliminates the hydrogen bond with T-R57. **d** Tat binding with the Y88F/D206L/H547A mutant of DAT. The triple mutations D-Y88F/D-D206L/D-H547A on TAT-hDAT structure. Compared to the above double mutations, the addition of Y88F mutation also eliminates the hydrogen bond with T-K19

Based on the constructed outward-open hDAT-Tat complex structure in our previous studies (Midde et al. 2015; Yuan et al. 2015, 2016a) hydroxyl group of the Y88 side chain on DAT may form a hydrogen bond with K19 on Tat (Midde et al. 2015); whereas the side chain and backbone of H547 on DAT may form a hydrogen bond with R49 on Tat (Quizon et al. 2016). Single mutations on Y88F or H547A may result in the significant disruption of the binding between Tat and DAT (Midde et al. 2015; Quizon et al. 2016; Yuan et al. 2016b). The negatively charged side chain of D206 in DAT could form a hydrogen bond with the positively charged side chain of R57 in Tat (Fig. 1a and b). Therefore, the mutation of these residues (D206L or D381L) is expected to impair the hydrogen bond between DAT and Tat (Fig. 1) and attenuate the inhibitory effect of Tat on DAT activity. Furthermore, D206 is a solvent exposed residue in the extracellular side of the hDAT structure, which resides far away from the dopamine binding site on the transporter (Fig. 1). Therefore, D206L is unlikely to directly interfere with the binding of dopamine with DAT, making this residue an ideal target for interfering with Tat-DAT binding without disrupting transporter function. Y88, D206, and H547 independently interact with Tat (Fig. 1b), suggesting that the combination of these mutants may additively result in greater interference to Tat-DAT binding. As shown in Fig. 1c, two hydrogen bonds between Tat and DAT were eliminated by D206L/H547A, while Fig. 1d shows that three hydrogen bonds were eliminated by Y88F/D206L/H547A.

### Tat Mutant K19A Attenuates Tat-induced Inhibition of DA Transport

**Fig. 2:**
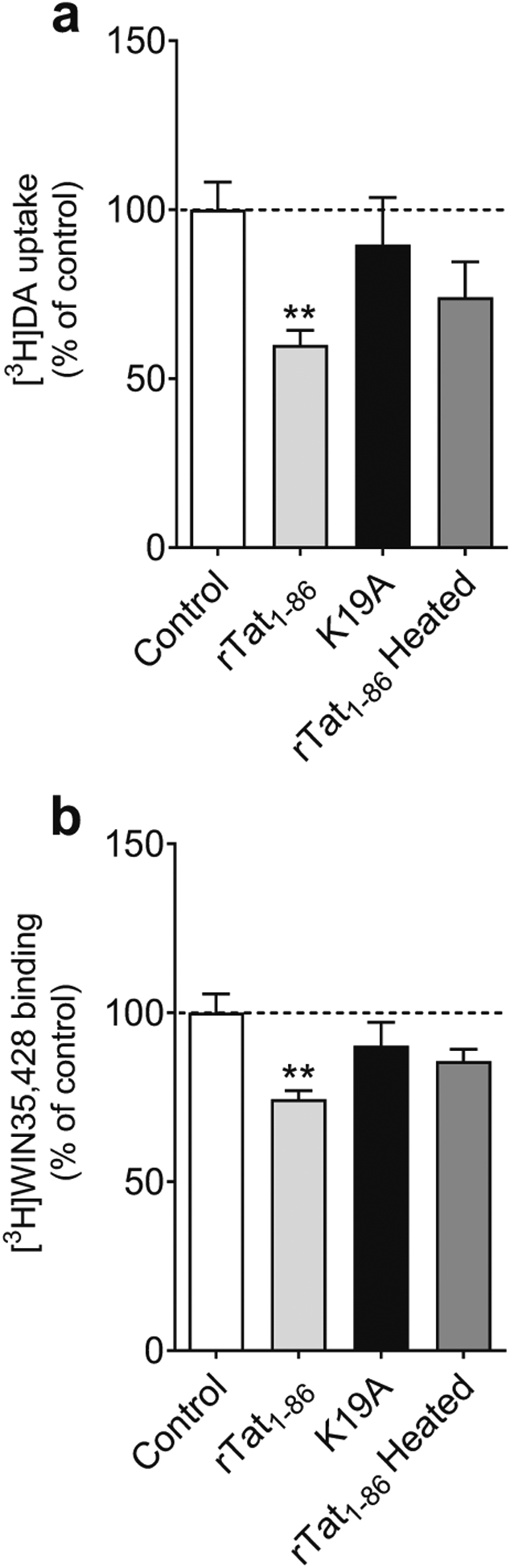
Disruption of the direct interaction between Tat and DAT by mutated Tat_1 – 86_. **a** Mutation of wild-type recombinant Tat_1 – 86_ (WT rTat_1 – 86_) at Lys19 (K19A) attenuates WT rTat_1 – 86_-induced inhibition of DA uptake. PC12 cells transiently transfected with WT hDAT were preincubated with or without 140 nM WT rTat_1 – 86_, K19A or heated rTat_1 – 86_ at room temperature for 20 min followed by addition of 5 nM [^3^H]DA. Specific [^3^H]DA uptake was determine in the presence of 10 μM nomifensine and desipramine. Heated rTat_1 – 86_ was used as a negative control. **b** K19A mutant attenuates WT rTat_1 – 86_-induced inhibition of DAT binding site. PC12 cells transiently transfected with WT hDAT were preincubated with 5 nM [^3^H]WIN35,428 on ice for 2 h in the presence or absence of 140 nM WT rTat_1 – 86_, K19A or heated rTat_1 –86_. Specific [^3^H]WIN35,428 binding was determine in the presence of 30 μM cocaine. Heated rTat_1 – 86_ was used as a negative control. Data are expressed as means from four independent experiments ± S.E.M. ** *p* < 0.01, compared to control (in the absence of Tat)

We have previously reported that Tyr88 on hDAT interacts with Tat residue K19 and that mutated Tyr88 (Y88F) attenuates Tat-induced inhibition of DA transport (Midde et al. 2015; Yuan et al. 2015, 2016a). This study further tested the DAT Y88-Tat K19 interaction by mutated K19 residue (K19A). Addition of 140 nM WT recombinant Tat_1 – 86_ (rTat_1 – 86_) decreased [^3^H]DA uptake (59.97 ± 4.30 % remaining, *t*_*(6)*_ = 4.34, *p* < 0.01, Fig. 2a) and [^3^H]WIN35,428 binding (74.41 ± 2.59 % remaining, *t*_*(6)*_ = 4.16, *p* < 0.01, Fig. 2b) in WT hDAT relative to their respective untreated controls (100 %) , whereas neither [^3^ H] DA uptake nor [^3^H]WIN35,428 binding was altered in the presence of heated rTat_1 – 86_. The WT rTat_1 – 86_-induced decrease in [^3^H]DA uptake and [^3^H]WIN35,428 binding was attenuated by K19A, indicating mutated Tat K19 residue disrupts the DAT-Tat interaction.

### Mutants on hDAT Differentially Alter DA Uptake Kinetics and DAT Binding

**Fig. 3:**
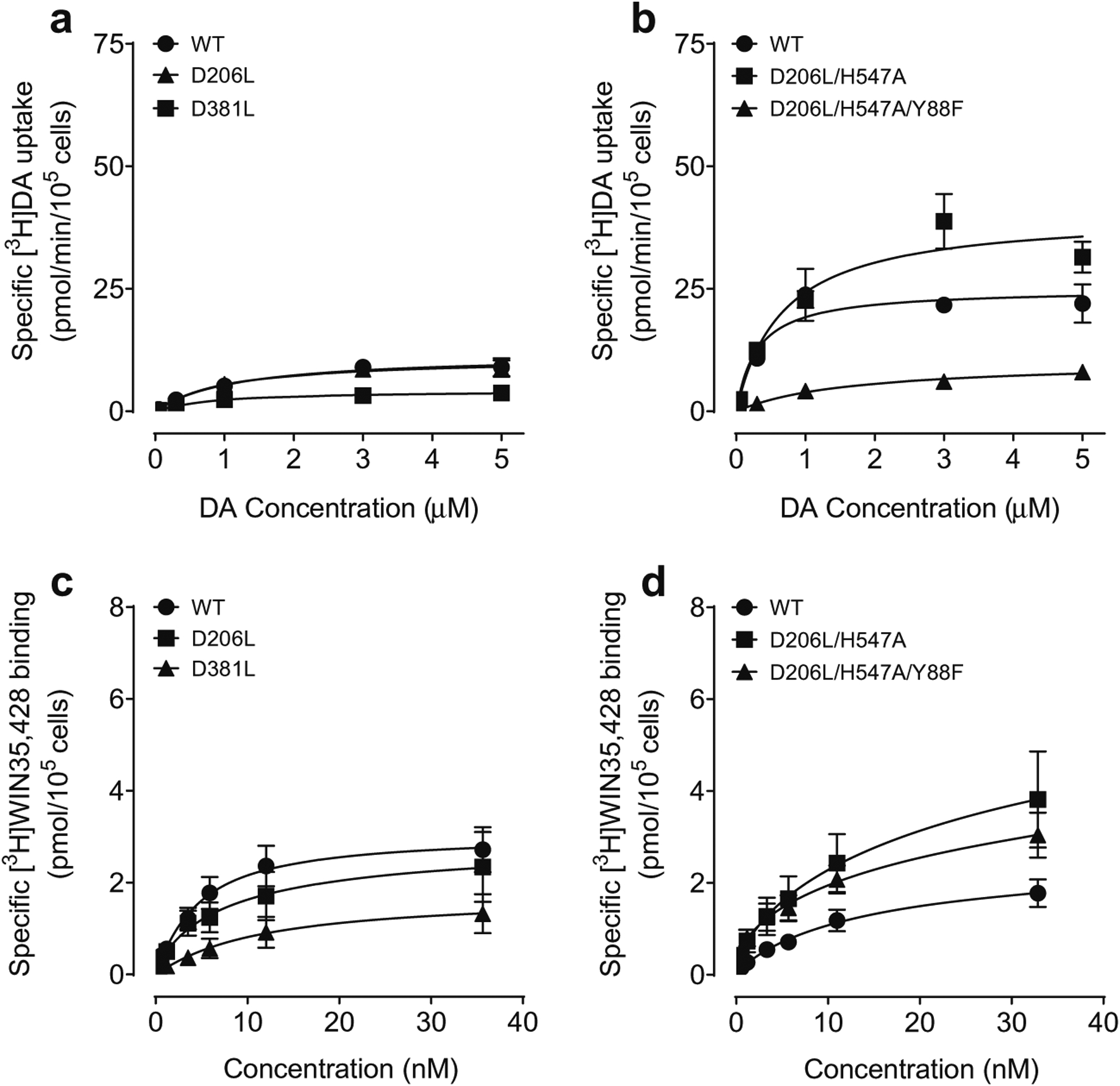
Kinetic parameters of [^3^H]DA uptake and [^3^H]WIN35,428 binding in WT hDAT and mutants. The specific [^3^H]DA uptake was determined in intact PC12 cells expressing WT hDAT (WT), D206L, D381L (**a**) and D206L/H547A, Y88F/D206L/H547A (**b**) using six concentrations of DA (0.03–5.0 μM) mixed with a fixed concentration of [^3^H]DA (500,000 dpm/well, specific activity: 21.2 Ci/mmol). In parallel, nonspecific uptake of each concentration of [^3^H]DA in the presence of 10 μM nomifensine and desipramine were subtracted from total uptake for calculating the specific DAT-mediated uptake. The V_*max*_ and K_*m*_ values were calculated by fitting the data to the Michaelis-Menten equation and represent the means from five to ten independent experiments ± S.E.M. * *p* < 0.05 compared to the respective WT hDAT. Saturation binding of [^3^H]WIN35,428 was tested in intact PC12 cells transfected with WT hDAT, D206L, D381L (**c**) and WT, D206L/H547A, Y88F/D206L/H547A (**d**) that were incubated with six concentrations of [^3^H]WIN35,428 (0.5–30 nM) on ice for 2 h. In parallel, nonspecific binding of each concentration of [^3^H]WIN35,428 in the presence of 30 μM cocaine was subtracted from total binding for calculating the specific DAT binding sites

To determine whether the mutations of Asp206 and Asp381 on hDAT influence basal DAT function, kinetic analysis of [^3^H]DA uptake was performed on WT hDAT, D206L and D381L. As shown in Table 1; Fig. 3a, compared to WT hDAT (12.43 ± 2.50 pmol/min/10^5^ cells), the V_max_ values were decreased in D381L (4.54 ± 0.95 pmol/min/10^5^ cells, *t*_(8)_ = 2.95, *p* < 0.05) and not altered in D206L (13.09 ± 3.55 pmol/min/10^5^ cells). Both mutants did not alter K_m_R values. As shown in Table 2; Fig. 3b, compared to WT hDAT (26.62 ± 3.87 pmol/min/10^5^ cells), the V_max_ values were increased in D206L/H547A (40.82 ± 3.60 pmol/min/10^5^ cells, *t*_(6)_ = 3.12, *p* < 0.05) and decreased in Y88F/D206L/H547A (10.78 ± 0.98 pmol/min/10^5^ cells, *t*_(6)_ = 3.97, *p* < 0.01) respectively. Compared to WT hDAT (0.45 ± 0.21 μM), K_m_ value was increased in Y88F/D206L/H547A (1.92 ± 0.37 μM, *t*_(6)_ = 3.45, *p* < 0.05) but not altered in D206L/H547A (0.69 ± 0.10 μM).

We assessed kinetic analysis of [^3^H]WIN35,428 binding in intact PC12 cells transfected with WT hDAT and mutants. As shown in Table 3; Fig. 3c, compared to WT hDAT (3.32 ± 0.58 pmol/10^5^ cells), the maximal binding sites (B_max_) of [^3^H]WIN35,428 were not altered in D206L (2.87 ± 0.96 pmol/10^5^ cells) and D381L (2.00 ± 0.63 pmol/10^5^ cells) (*ps* > 0.05); however, the K_d_ values of D381L significantly increased (17.52 ± 6.57 nM, *t*_(16)_ = 2.50, *p* < 0.05) in comparison to WT hDAT (5.98 ± 0.73 nM). As shown in Table 4; Fig. 3d, although the B_max_ values of [^3^H]WIN35,428 were not altered in D206L/H547A and Y88F/D206L/H547A relative to WT hDAT, the K_d_ value was significantly decreased in Y88F/D206L/H547A (6.90 ± 1.26 nM, *t*_(6)_ = 2.48, *p* < 0.05) in comparison to WT hDAT (12.53 ± 1.88 pmol/min/10^5^ cells).

**Fig. 4:**
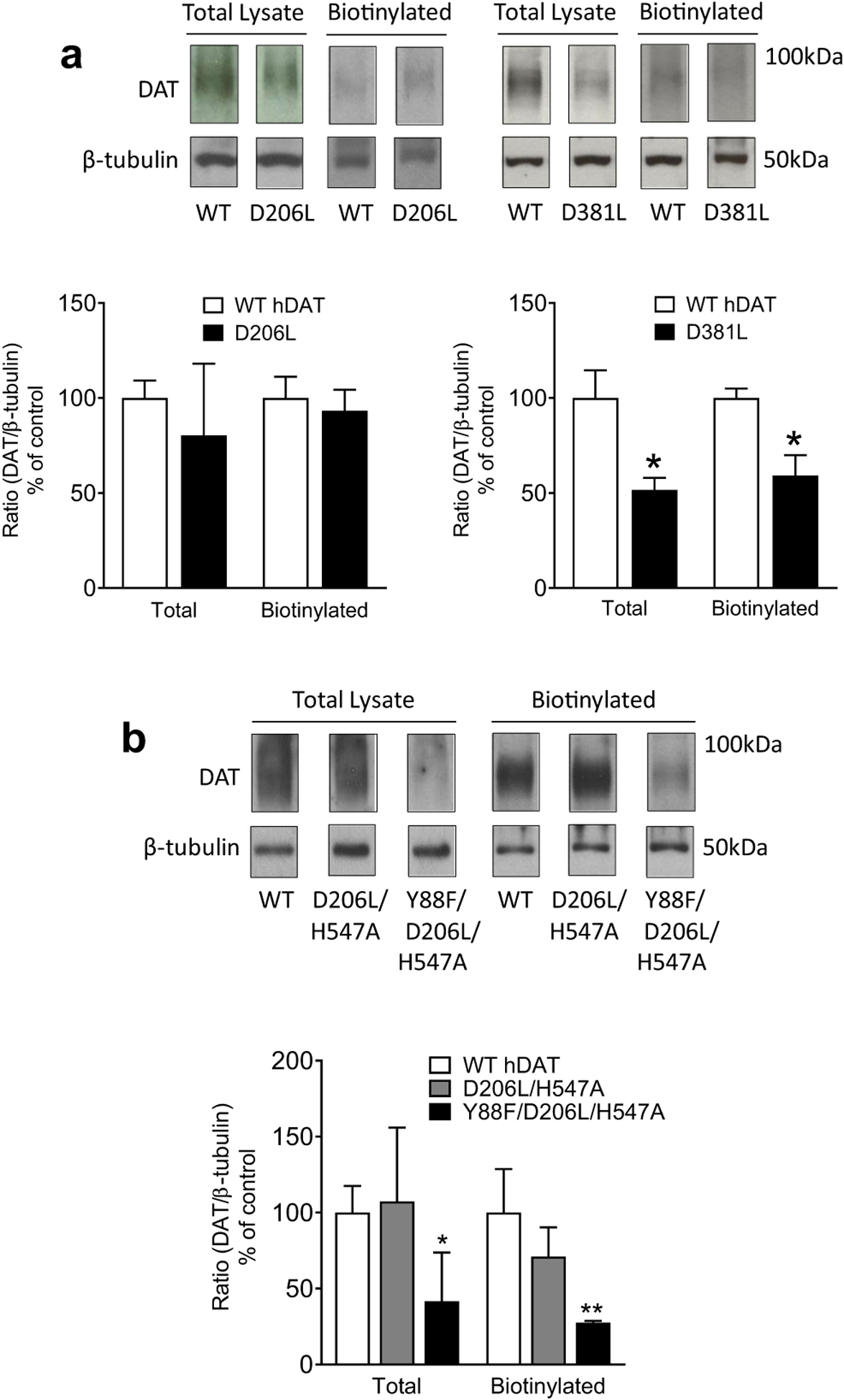
DAT surface expression in WT hDAT and mutants. DAT surface expression of WT hDAT and mutants was determined by biotinylation assay followed by Western blotting. **a** top panels: representative immunoblots of total (Total Lysate) and cell surface (Biotinylated) fraction of DAT in WT hDAT, D206L, and D381L mutants. β-tubulin was used as control protein for sample loading. Bottom panels: the quantification of total and cell surface expression of DAT was expressed as means ± S.E.M of the ratio of total or biotinylated DAT immunoreactivity to β-tubulin immunoreactivity (n = 4–5). *p < 0.05, compared to WT hDAT. **b** top panels: representative immunoblots of total (Total Lysate) and cell surface (Biotinylated) fraction of DAT in WT hDAT, D206L/H547A, and Y88F/D206L/H547A mutants. Bottom panels: the quantification of total and cell surface expression of DAT was expressed as means ± S.E.M of the ratio of total or biotinylated DAT immunoreactivity to β-tubulin immunoreactivity (n = 4–5). All raw immunoblots are provided in supplementary material. *p < 0.05, **p < 0.01 compared to WT hDAT

To determine whether mutations of hDAT alter subcellular distribution of DAT, biotinylation followed by immunoblot assays were performed in both total and cell surface (biotinylated) fractions from PC12 cells transiently transfected with either WT hDAT or mutants (Fig. 4). Neither total nor surface DAT was altered in D206L relative to WT hDAT, which corresponds to the unaltered V_max_ values as shown in Fig. 3a. Compared to controls, total DAT and surface DAT in D381L were decreased by 48 % (*t*_*(8)*_ = 3.04, *p* < 0.05) and 41 % (*t*_*(8)*_ = 3.44, *p* < 0.05), respectively, which correspond to the decreased V_max_ in D381L shown in Table 1; Fig. 3a. Interestingly, although the increased V_max_ was observed in D206L/H547A, this mutant did not alter total and surface DAT relative to WT hDAT (Fig. 4b). The DAT expression in Y88F/D206L/H547A was significantly decreased by 58 % in total (*t*_(4)_ = 2.76, *p* < 0.05) and 73 % in surface (*t*_(4)_ = 4.38, *p* < 0.05) fractions, respectively, compared to WT hDAT, which corresponds to the significant decrease in V_max_ (Table 2; Fig. 3b).

### DAT Mutants Alter the Inhibition Potency of DA Uptake and DAT Binding by Substrates and Inhibitors

As shown in Table 1, compared to WT hDAT (1809 ± 493 nM), D381L decreased IC_50_ values for DA inhibiting [^3^H]DA uptake (338 ± 39 nM, *t*_*(5)*_ = 2.80, *p* < 0.05), while D206L did not alter the IC_50_ value. In addition, both D206L and D381L did not alter the IC_50_ values for cocaine and GBR12909 relative to WT hDAT. As shown in Table 3, D206L and D381L did not alter the IC_50_ values for DA inhibiting [^3^H]WIN35,428 binding; however, D381L increased the IC_50_ values for cocaine (1480 ± 276, *t*_*(8)*_ = 5.70, *p* < 0.001) and GBR12909 (2620 ± 404, *t*_*(8)*_ = 3.50, *p* < 0.01) compared to WT hDAT (308 ± 55 and 770 ± 193, respectively). As shown in Table 2, compared to WT hDAT, D206L/H547A preserved potency for DA, cocaine, or GBR12909 inhibiting [^3^H]DA uptake, while Y88F/D206L/H547A increased IC_50_ values for DA (1830 ± 587, *t*_*(9)*_ = 2.53, *p* < 0.05) and decreased IC_50_ values for GBR12909 (180 ± 18, *t*_*(8)*_ = 5.58, *p* < 0.001) compared to WT hDAT (467 ± 85 and 660 ± 84 nM, respectively). Both double and triple mutants did not alter the IC_50_ values for cocaine.

### D206L, D206L/H547A, and Y88F/D206L/H547A Attenuate Tat-induced Inhibition of DA Transport

**Fig. 5:**
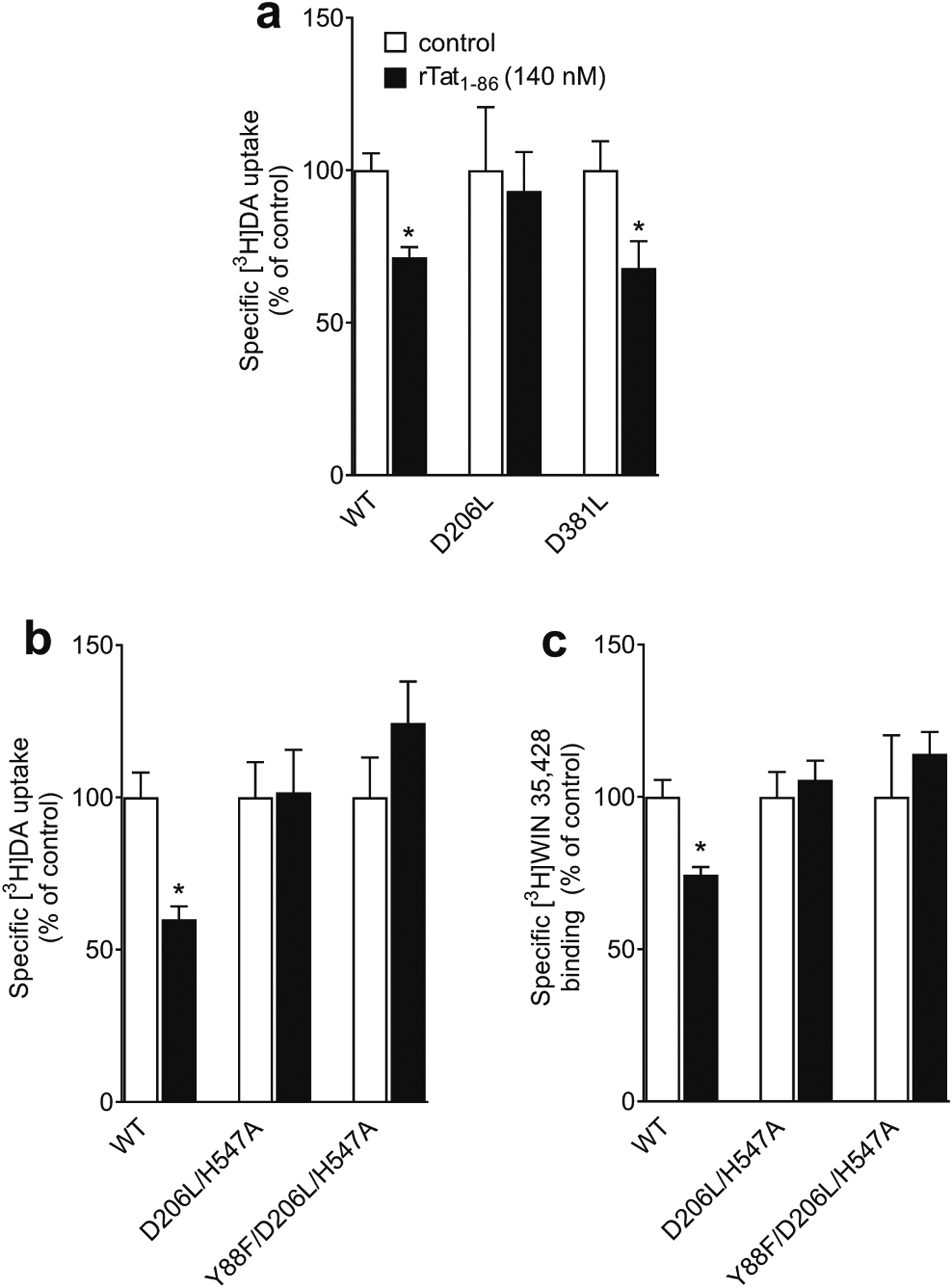
Mutational effect of hDAT on Tat-induced inhibition of [^3^H]DA uptake and [^3^H]WIN35,428 binding. **a**, **b** PC12 cells expressing WT hDAT (WT), D206L, D381L, D206L/H547A, or Y88F/D206L/H547A mutants were preincubated with or without recombinant Tat_1 – 86_ (rTat_1 –86_, 140 nM) at room temperature for 20 min followed by the addition of 5 nM [^3^H]DA. In parallel, nonspecific uptake (in the presence of 10 μM nomifensine and desipramine) was subtracted from total uptake to calculate DAT-mediated uptake. **c** PC12 cells expressing WT, D206L/H547A or Y88F/D206L/H547A mutants were incubated with or without recombinant Tat_1 – 86_ (rTat_1 – 86_, 140 nM) and [^3^H]WIN35,428 on ice for 2 h. In parallel, nonspecific binding (in the presence of 30 μM cocaine) was subtracted from total binding to calculate specific binding. Data are presented as a percentage of untreated control per group, expressed as means ± S.E.M (n = 4). *p < 0.05, compared to the percentage of control (in the absence of Tat)

Based on the prediction of computational modeling (Fig. 1), either single mutants (D206L or D381L) or the combination of mutants (D206L/H547A or Y88F/D206L/H547A) would interfere with the hydrogen bonds between Tat and DAT, thereby affecting Tat-induced inhibition of DA transport. This study validated the predictions by determining the specific [^3^H]DA uptake and [^3^H]WIN35,428 binding in the presence or absence of 140 nM rTat_1 − 86_. As shown in Fig. 5a, addition of rTat_1 − 86_ decreased DA uptake by 29 % in WT hDAT (*t*_(6)_ = 4.39, *p* < 0.01) and by 32 % in D381L (*t*_(6)_ = 2.47, *p* < 0.05), while Tat did not alter DA uptake in D206L relative to the respective controls. In a separate study, the addition of 140 nM rTat_1 – 86_ resulted in a 40 % decrease in [^3^H]DA uptake in WT hDAT (Fig. 5b, *t*_(6)_ = 4.34, *p* < 0.01) and a 26 % reduction in [^3^H]WIN35,428 binding (Fig. 5c, *t*_(6)_ = 4.16, *p* < 0.01), respectively, which was attenuated in D206L/H547A or Y88F/D206L/H547A.

### Mutational Effects of hDAT on Zinc-induced Transporter Conformational Transition and Basal DAT-mediated Efflux

**Fig. 6:**
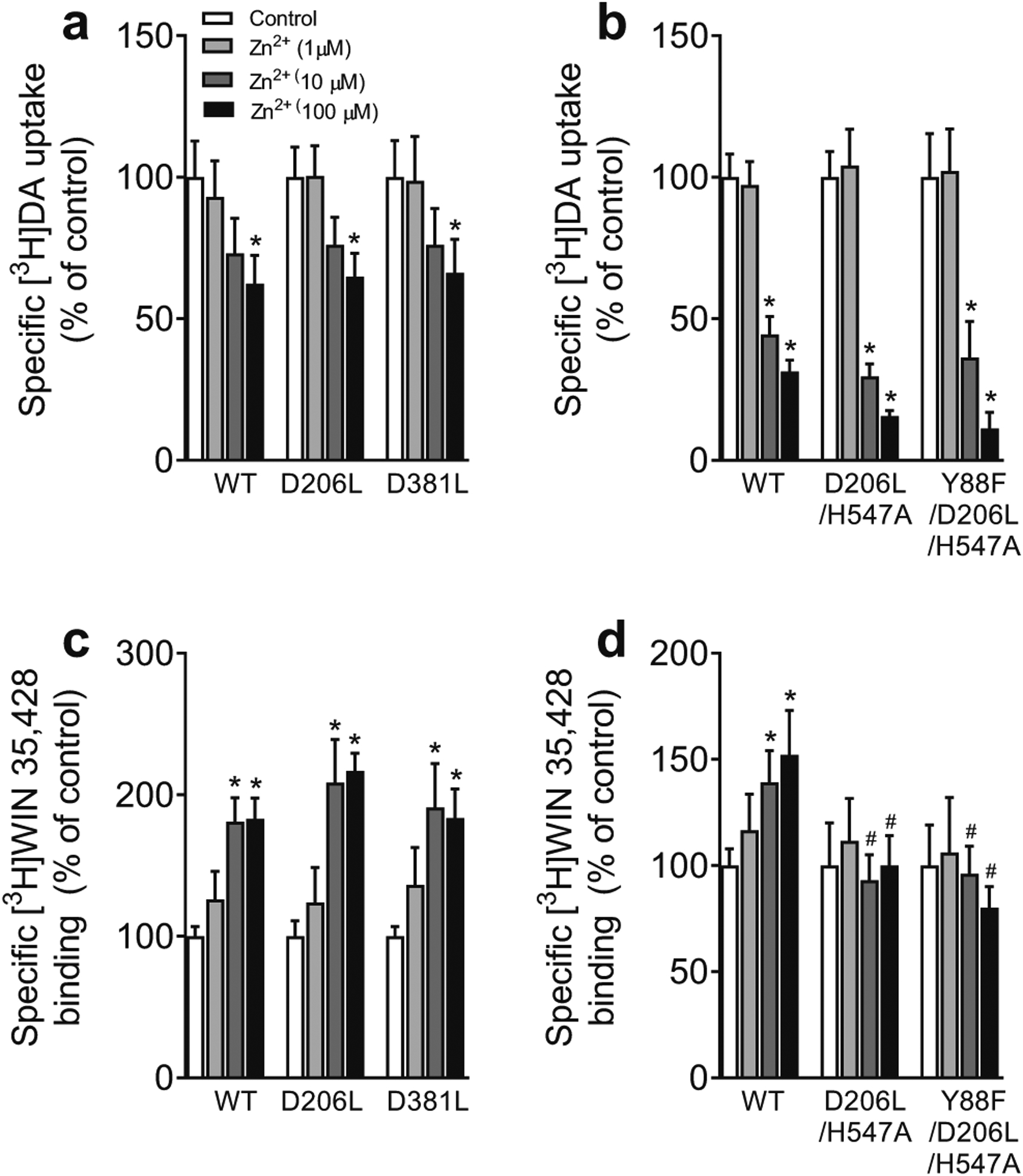
Mutational effect of hDAT on zinc regulation of [^3^H]DA uptake and [^3^H]WIN35,428 binding. PC12 cells transiently expressing WT hDAT (WT) or mutants were incubated with buffer alone (control) or three concentrations of ZnCl_2_ (1, 10, or 100 μM) followed by [^3^H]DA uptake or [^3^H]WIN 35,428 binding. **a** Mutants D381L and D206L do not affect zinc-mediated decrease in [^3^H]DA uptake. Two-way ANOVA analysis on the specific [^3^H]DA uptake in WT, D381L, and D206L revealed a significant main effect of mutation (F_(2,17)_ = 14.92, *p* < 0.001), zinc (F_(1,17)_ = 125.86, *p* < 0.05), and a significant mutation × zinc interaction (F_(2,17)_ = 15.97, *p* < 0.001). **b** Mutants D381L and D206L do not affect zinc-mediated decrease in increase in [^3^H]WIN 35,428 binding. Two-way ANOVA analysis revealed a significant main effect of mutation (F_(2, 9)_ = 48.9, *p* < 0.001), zinc (F_(1, 9)_ = 167.9, *p* < 0.05), and a significant mutation × zinc interaction (F_(2, 9)_ = 38.2, *p* < 0.001). **c** Both D206L/H547A and Y88F/D206L/H547A do not alter zinc-induced decrease in DA uptake. Two-way ANOVA analysis in WT, D206L, and D381L revealed a significant main effect of mutation (F_(2, 11)_ = 28.81, *p* < 0.001), zinc (F_(1, 11)_ = 140.30, *p* < 0.001), and a significant mutation × zinc interaction (F_(2, 11)_ = 23.47, *p* < 0.001). **d** The zinc-mediated increase in [^3^H]WIN binding in WT hDAT but not in D206L/H547A and Y88F/D206L/H547A. There were significant main effects of mutation (F_(2, 12)_ = 4.33, *p* < 0.05) and a significant mutation × zinc interaction (F_(2, 12)_ = 6.43, *p* < 0.05). The graphs illustrate specific [^3^H]DA uptake and [^3^H]WIN 35,428 binding expressed as mean ± S.E.M. of the respective controls set to 100 % for mutants. *p < 0.05, compared to the respective controls. ^#^ *p* < 0.05, compared to WT hDAT within same zinc concentration. (n = 4–7)

WT hDAT contains endogenous zinc (Zn^2+^) binding sites that serve to stabilize the transporter in the outward-facing conformation when bound to Zn^2+^. This promotes DA binding (thereby increasing [^3^H]WIN35,428 binding) while inhibiting DA translocation (decreasing [^3^H]DA uptake)(Norregaard et al. 1998; Loland et al. 2003). The addition of Zn^2+^ partially reverses the transporter from the inward-facing state to the outward-facing state, making this technique useful for determining alterations to the transporter’s conformational equilibrium. This study examined the zinc modulation of [^3^H]DA uptake and [^3^H]WIN35,428 binding in WT hDAT and its mutants. Addition of Zn^2+^ resulted in a similar decrease in [^3^H]DA uptake observed in single mutants (Fig. 6a, D206L or D381L) and multiple mutants (Fig. 6b, D206L/H547A and Y88F/D206L/H547A) in a concentration-dependent manner, indicating that these mutants do not alter zinc-mediated DA transport.

With regard to zinc-mediated [^3^H]WIN35,428 binding in WT hDAT and mutants (Fig. 6c), addition of Zn^2+^ resulted in an increase in the specific [^3^H]WIN35,428 binding in WT hDAT, D206L, and D381L in a concentration-dependent manner. These results indicate that neither D206L nor D381L mutant alters the zinc-mediated [^3^H]WIN35,428 binding relative to WT hDAT. Figure 6d shows that the specific [^3^H]WIN35,428 binding in WT hDAT was increased at 10 μM Zn^2+^ by 39 % (*t*_(8)_ = 2.25, *p* < 0.05) and at 100 μM Zn^2+^ by 52 % (*t*_(8)_ = 2.30, *p* < 0.05), respectively, which was attenuated in D206L/H547A or Y88F/D206L/H547A.

**Fig. 7:**
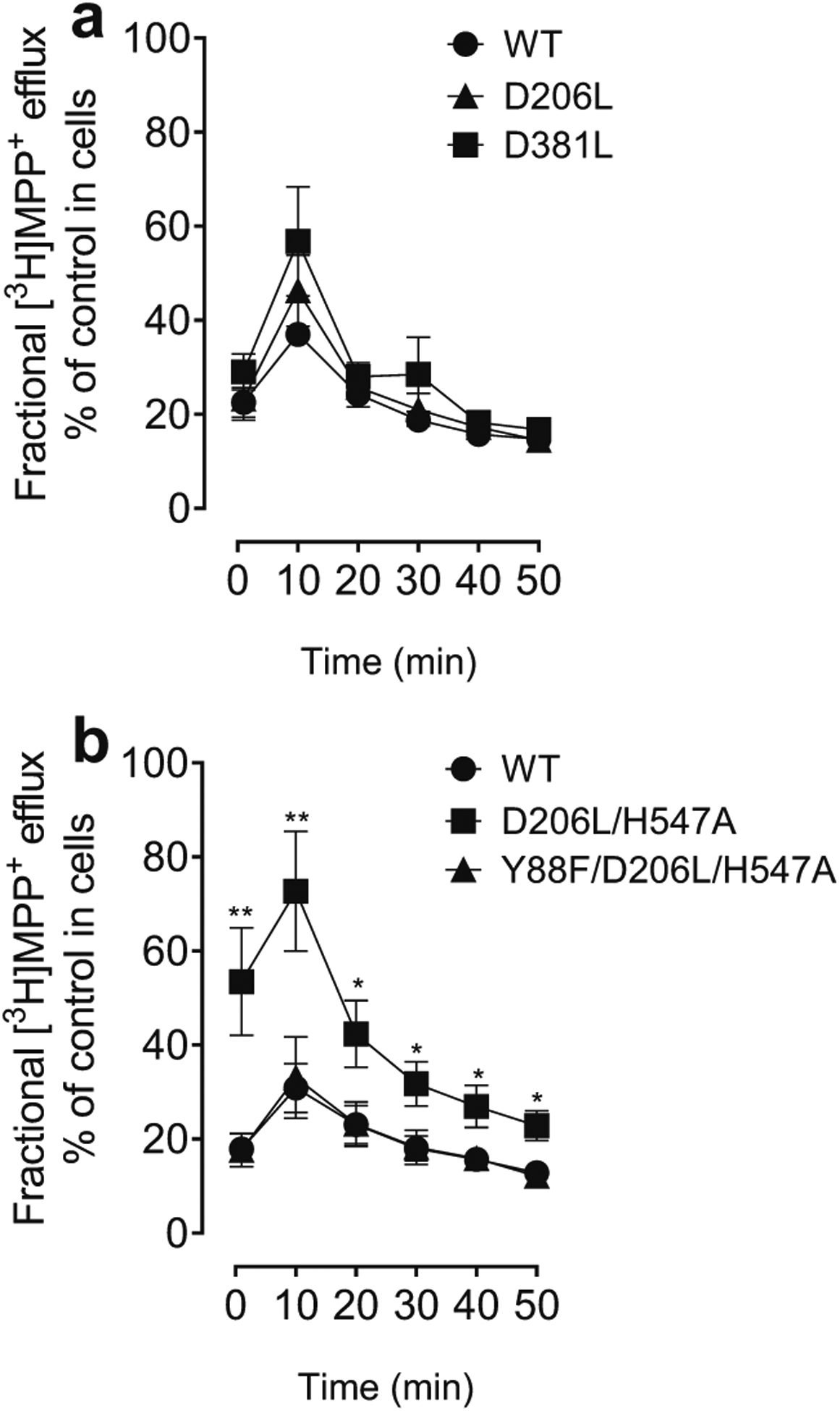
Mutational effect of hDAT on functional efflux of MPP^+^. PC12 cells transfected with WT hDAT or mutants were preincubated with assay buffer containing 5 nM [^3^H]MPP^+^ at room temperature for 20 min. After incubation, cells were washed and incubated with fresh buffer at indicated time points. Subsequently, the buffer was removed, and radioactivity in the buffer and residual radioactivity in the cells was counted. Each fractional efflux of [^3^H]MPP^+^ in WT hDAT or mutants was expressed as a percentage of total [^3^H]MPP^+^ in the cells at the start of the experiment. Fractional [^3^H]MPP^+^ efflux levels at 1, 10, 20, 30, 40 and 50 min are expressed as a percentage of total [^3^H]MPP^+^ with preloading with 0.005 μM introduced to the cells at the start of the experiment. **a** Two-way ANOVA on functional MPP^+^ efflux of WT hDAT, D206L and D381L revealed a significant main effect of time (F_(1, 9)_ = 53.10, *p* < 0.001). **b** Two-way ANOVA analysis on the fractional efflux levels of [^3^H]MPP^+^ in WT hDAT, D206L/H547A and Y88F/D206L/H547A revealed a main effect of genotype (F_(2, 21)_ = 6.96, *p* < 0.01), time (F_(1, 21)_ = 36.26, *p* < 0.001) and significant interaction of genotype × time (F_(2, 21)_ = 8.33, *p* < 0.01) * *p* < 0.05, ** *p* < 0.01 compared to WT hDAT. (n = 4–8)

To further determine the mutational effects of hDAT on transporter conformational transitions, we determined the mutational effects of hDAT on the fractional efflux levels of [^3^H]MPP^+^ in WT hDAT and its mutants. Neither D206L nor D381L altered the MPP^+^ efflux across the indicated times compared to the respective WT hDAT (Fig. 7a). As shown in Fig. 7b, while Y88F/D206L/H547A did not alter the MPP^+^ efflux levels relative to WT hDAT, the MPP^+^ efflux levels in D206L/H547A were elevated at all indicated time (*ps* < 0.05, Bonferroni *t*-test) compared to WT hDAT, indicating D206L/H547A induces transporter conformational transitions.

### H547A Mutant Renders the Transporter Into a More Outward-facing Conformation and Alters Basal Palmitoylation

**Fig. 8:**
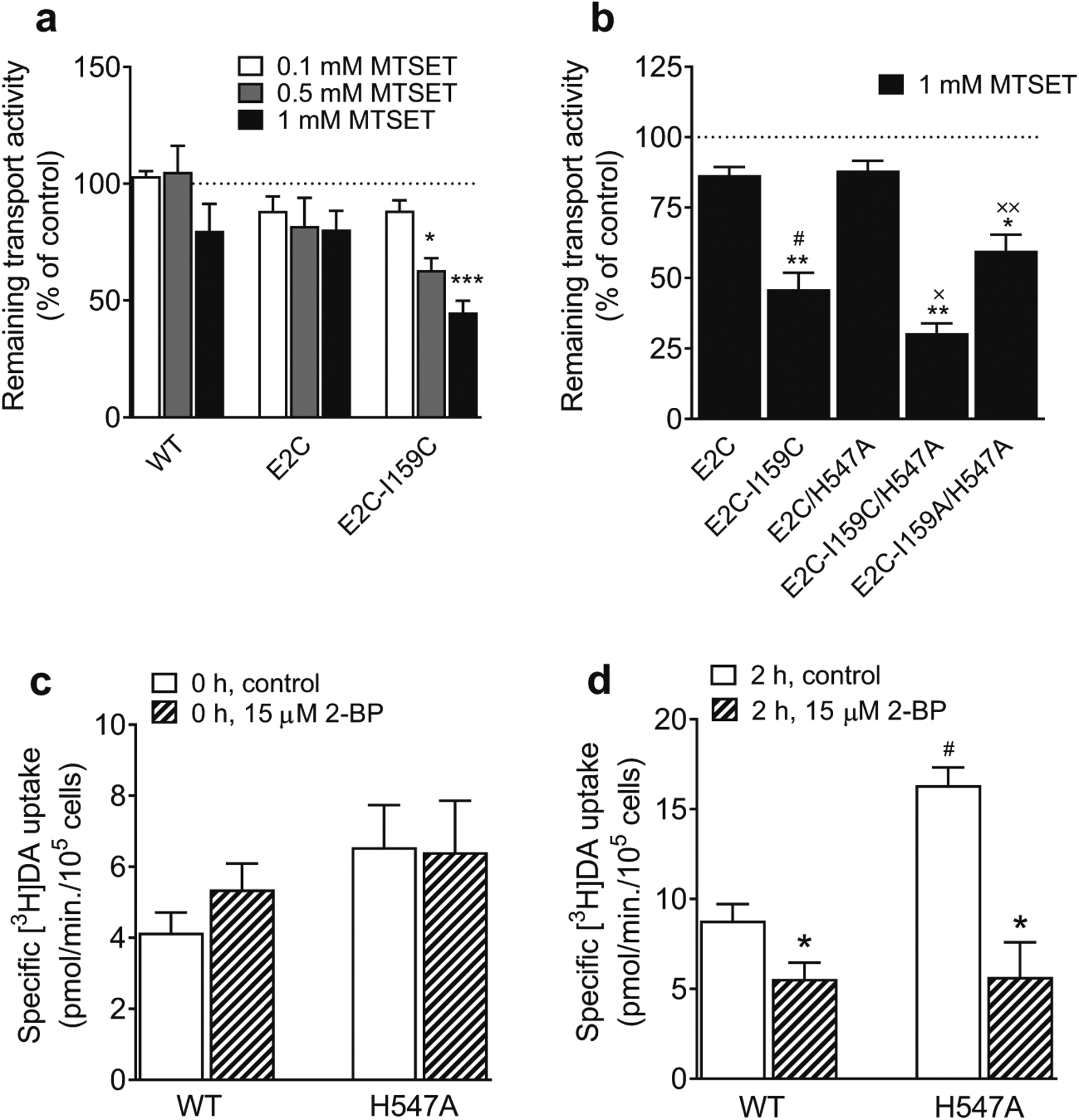
H547A renders an inserted cysteine more reactive to MTSET inactivation and alters basal palmitoylation compared to WT hDAT. HEK293 cells transiently expressing WT hDAT (WT) or E2C hDAT constructs were treated with a range of concentrations (0.1, 0.5, 1.0 mM) of MTSET for 10 min followed by the addition of 5 nM [^3^H]DA for 8 min. **a** the concentration-dependent effects of MTSET on DA uptake. Two-way ANOVA analysis revealed no significant main effects of genotype and their interaction on MTSET-mediated DA uptake, a significant main effect of MTSET (F_(1, 6)_ = 35.55, *p* < 0.01) was found. (*p < 0.05, ***p < 0.001; n = 4). **b** Effects of 1 mM MTSET on DA uptake in E2C, E2C-I159C, E2C/H547A, E2C-I159C/H547A, and E2C-I159A/H547A. Two-way ANOVA analysis on MTSET-mediated [^3^H]DA uptake in positive control E2C-I159C/H547A and negative control E2C-I159A/H547A revealed a significant main effects of genotype (F_(4, 40)_ = 139.67, *p* < 0.001) and treatment (F_(1, 40)_ = 40.03, *p* < 0.001), and genotype × treatment interaction (F_(4, 40)_ = 2.62, *p* < 0.05). (* *p* < 0.05, ** *p* < 0.01 compared to the respective controls). # *p* < 0.001, compared to E2C; ^×^ *p* < 0.05 compared to E2C-I159C; ^××^ *p* < 0.05 compared to E2C-I159C/H547A, (n = 5). Effects of 2-BP on V_max_ of [^3^H]DA uptake at zero (**c**) or two-hour (**d**) time point in WT hDAT and H547A in the presence or absence of 15 μM 2-BP. Two-way ANOVA on the V_max_ values with or without 2-BP revealed significant main effects of genotype (F_(1,10)_ = 10.3, *p* < 0.01) and treatment (F_(1,10)_ = 34.2, *p* < 0.001) as well as a significant interaction of genotype × treatment (F_(1,10)_ = 9.7, *p* < 0.05). * *p* < 0.05 compared to control; ^#^ *p* < 0.05 compared to WT hDAT within control group, (n = 3–4)

Our data show that the V_max_ was not altered in D206L but increased in H547A (Quizon et al. 2016). To explore potential conformational changes in H547A, we utilized an assay to test the accessibility to the reactivity of cysteine inserted into position 159 (Cys159) in transmembrane 3 of hDAT. This accessibility of Cys159 is based on whether the extracellular gate is open or closed. For example, when the extracellular gate is open, Cys159 becomes accessible to the extracellular environment, whereas it is inaccessible when the gate is closed (Loland et al. 2004). The reaction of the cysteine to MTSET could inactivate the transporter, which allow us to use [^3^H]DA uptake as a functional readout for a mutation of hDAT at I159 (isoleucine to cysteine, I159C) reactivity. The I159C mutant was inserted into DAT background (E2C) in which the two external endogenous cysteines were mutated to alanines (C90A-C306A), resulting in DAT E2C I159C. Previous studies show that [^3^H]DA uptake in cells expressing E2C I159C was inhibited in the presence of MTSET, whereas no inhibition of DA uptake was observed in E2C (Loland et al. 2004, 2008), suggesting a stabilization of the transporter in a conformation open to the extracellular environment. Based on this theory, we hypothesized that the insertion of H547A into E2C I159C results in a significant increase in MTSET-induced inhibition of DA uptake. A pilot study shows that MTSET concentration-dependently inhibited [^3^H]DA uptake in DAT E2C-I159C, while MTSET had no effect on [^3^H]DA uptake in DAT E2C-hDAT (Fig. 8a). In particular, the [^3^H]DA uptake by E2C-I159C was inhibited by the application of 1 mM MTSET by 55 % (*t*_(2)_ = 11.13, *p* < 0.05) relative to no MTSET addition. Next, we tested the effect of H547A mutant on the E2C-I159C reactivity (Fig. 8b). The insertion of H547A into E2C or E2C-I159C (E2C/H547A, E2C-I159C/H547A or E2C-I159A/H547A) displayed a differential reaction to 1 mM MTSET. First, both E2C and E2C/H547A showed no effect of MTSET on DA uptake, suggesting that addition of H547A mutant does not alter DAT E2C control. Second, compared to E2C control, [^3^H]DA uptake was inhibited in E2C-I159C (*t*_(8)_ = 6.34, *p* < 0.001). Third, DA uptake was inhibited by 65 % in E2C-I159C/H547A (30.4 ± 3.52, *t*_(8)_ = 5.24, *p* < 0.001) and 31 % in E2C-I159A/H547A (59.7 ± 5.7, *t*_(8)_ = 2.17, *p* < 0.05) relative to E2C (86.5 ± 6.0). Moreover, compared to E2C-I159C (46.0 ± 6.0), [^3^H]DA uptake was further inhibited by 34 % in E2C-I159C/H547A (30.4 ± 3.5, *t*_(8)_ = 8.93, *p* < 0.001), which was reversed in the negative control E2C-I159A/H547A (59.7 ± 5.7, *t*_(8)_ = 2.92, *p* < 0.05), suggesting that mutation of His547 may change the transporter conformational state toward an outward open conformation.

Our previous study demonstrates that the H547A-induced increase in V_max_ is mediated by alterations in basal PKC activity (Quizon et al. 2016). Recent studies reported that DAT kinetics is also regulated by a reciprocal phosphorylation and palmitoylation mechanism (Moritz et al. 2015; Foster and Vaughan 2017; Rastedt et al. 2017). To determine whether the H547A-induced increase in DA uptake is a result of alteration of basal palmitoylation levels, we determined the kinetic parameters (V_max_ and K_m_) of DA uptake in WT hDAT and H547A in the presence or absence of 2-bromopalmitate (2-BP), a palmitoylation inhibitor. As shown in Table 5; Fig. 8c and d, a pilot study was designed to determine the time-dependent inhibitory effect of 2-BP on DA uptake, in which at zero time point, 2-BP had no inhibitory effect on V_max_ or K_m_ in WT hDAT and H547A relative to controls (Fig. 8c and d). However, at two-hour time point after addition of 2-BP, compared to the respective controls (in the absence of 2-BP) for WT hDAT (8.80 ± 0.92) and H547A (16.33 ± 0.99), addition of 15 μM 2-BP decreased the V_max_ in WT hDAT (5.55 ± 0.92 remaining, *t*_(6)_ = 2.50, *p* < 0.05) and H547A (5.56 ± 1.93 remaining, *t*_(6)_ = 4.92, *p* < 0.01). Within the vehicle groups, the V_max_ was increased in H547A compared to WT hDAT (*t*_(5)_ = 5.51, *p* < 0.01). Additionally, 2-BP at zero or two-hour time point did not alter the K_m_ values, whereas H547A mutant increased the K_m_ values relative to WT hDAT (Table 5), which is consistent with our previous report (Quizon et al. 2016).

## Discussion

Our previous studies have demonstrated that HIV-1 Tat protein interacts with Y88 or H547 residue on hDAT and mutations of these residues result attenuation of Tat-induced inhibition of DA transport (Midde et al. 2015; Quizon et al. 2016). Particularly, mutations of Y88 (Y88F) and H547 (H547A) preserved and increased DA transport, respectively (Midde et al. 2015; Quizon et al. 2016). The current results show that mutation of a novel D206 residue on hDAT (D206L) preserved normal DA uptake and attenuated Tat-induced inhibition of DA uptake, which is similar to our previous observation in Y88F (Midde et al. 2015). These findings suggest that Y88 and D206 residues are important for stabilizing the compact structure of hDAT for DA transport and Tat binding to hDAT, which was supported by the prediction from our computational model of the hDAT-Tat complex. First, D206 is solvent exposed, which indicates that it is not involved in direct interaction with other parts of the hDAT. Therefore, our model predicts that mutating D206 residue may not disturb the hDAT transport process. Second, D206 residue is far away from the DA substrate binding site, indicating that the mutation of this residue is not likely to influence substrate (i.e., DA) binding. Third, D206 residue forms a hydrogen bond with T-R57 (which represents R57 of Tat), which indicates that the mutation of D206 is capable of interfering with hDAT and Tat binding, inducing attenuation of Tat effect. In contrast, D381L displayed a decrease in the V_max_ of DA uptake and the DA uptake potency, but increased the inhibitory potency of [^3^H]WIN35,428 binding for cocaine and GBR12909, suggesting that D381L alters the basal DA transport function. Moreover, D381L had no effect on Tat-induced inhibition of DA transport because D381L was only observed to bind to T-R57 with a relatively large distance compared to D206L. Thus, mutational effects of hDAT residues on Tat-inhibited DA transport largely depend on the functional relevance of these residues for Tat binding to hDAT.

Considering that Tat protein realistically binds to more than one recognition residue on hDAT, this study evaluated the effects of multiple mutations of these key recognition hDAT residues on basal DAT function as well as their ability to disrupt hDAT-Tat interaction. Studying multiple mutants has traditionally been utilized to uncover key mechanistic insights underlying monoamine transporter pharmacokinetics and function (Itokawa et al. 2000; Pedersen et al. 2014; Sun et al. 2019). The D206A/H547A mutant displayed a 53 % increase in the V_max_ of DA uptake, which partially retains the magnitude of increased DA uptake (195 %) in H547A observed in our previous study (Quizon et al. 2016), suggesting a critical role of the mutated H547 residue in regulating transporter activity. Interestingly, Y88F/D206L/H547A induced a 147 % reduction in the V_max_ of DA uptake, whereas neither Y88F (Midde et al. 2015) nor D206L altered the V_max_, suggesting no additive effects of the combination of the mutated residues on the basal DA transport. Moreover, Y88F/D206L/H547A increased the K_m_ of DA uptake, whereas the K_m_ was not altered in D206L in the current study or Y88F and H547 from our previous reports (Midde et al. 2015; Quizon et al. 2016). Furthermore, Y88F/D206L/H547A increased DA uptake potency for DA and GBR12909. The reduction of DA transport could be attributed to the reduction of total DAT protein or surface DAT expression (Zhu and Reith 2008). D381L displayed a significant reduction of total and surface DAT expression, suggesting the decreased V_max_ in D381L is due to changing total DAT protein expression. Interestingly, our current and previous results show that neither total DAT nor surface DAT expression was altered in Y88F, D206L or H547A (Midde et al. 2015; Quizon et al. 2016), however, an reduction of total and surface DAT was observed in Y88F/D206L/H547A. We have reported that mutated Lys92 (K92M) decreases DA uptake as well as total DAT protein (Midde et al. 2015). Moreover, Y88F/D206L/H547A did not alter the B_max_ of [^3^H]WIN35,428 binding but increased its binding potency. These results suggest that the triple mutations do not alter DAT binding sites but may decrease DAT protein expression. Thus, the current findings suggest that when these residues interact with Tat could disrupt the intermolecular interactions within DAT by allosteric modulation and transporter conformational transitions, leading an alteration in the binding site of substrate DA.

Through the integrated computational modeling and experimental validation, the current study further validated the hDAT-Tat complex model by mutating Lys19 (K19A) on Tat protein. Although we have previously demonstrated that mutation of Cys22 residue (C22G) on Tat attenuates Tat-induced inhibition of DA transport (Zhu et al. 2009), the underlying mechanism of C22G-induced attenuation of Tat inhibitory effect is a result of changing normal Tat protein structure folding rather than DAT-Tat interaction. The current results show that K19A attenuated the wild type Tat-induced reduction in both [^3^H]DA uptake and [^3^H]WIN35,428 binding, which strongly support the computationally predicted the DAT-Tat interaction. The WIN35,428 binding site shares the pharmacological identity with the substrate DA uptake carrier as well as cocaine binding domain (Pristupa et al. 1994; Reith and Coffey 1994). We have demonstrated that Tat allosterically interacts with DAT by dissociating cocaine-mediated [^3^H]WIN35,428 binding (Zhu et al. 2011), indicating that Tat protein does not overlap with DAT. Therefore, the mutated K19 could disrupt the DAT-Tat interaction, leading an attenuation of Tat-induced inhibition of DA uptake and DAT binding. On the other hand, according to our computational modeling prediction, the mutation of either Y88 or H547 residue on hDAT could independently interact with Tat residue K19 or R49 with a hydrogen bond (Yuan et al. 2016a), which have been validated by attenuating the inhibitory effect of Tat on DA transport in Y88F or H547A (Midde et al. 2015; Quizon et al. 2016). The current results show that D206L, D206L/H547A or Y88F/D206L/H547A attenuated Tat-induced inhibition of DA uptake and DAT binding, which further validate our computational prediction: two and three hydrogen bonds in the hDAT-Tat interface could be eliminated by the double and triple mutations of these residues. Thus, our findings will provide insight into identifying novel therapeutic targets for the treatment of DAT-mediated dysregulation of dopaminergic function.

Given that Tat modulates DAT function allosterically (Zhu et al. 2011), the current study determined the underlying mechanism of DAT mutant-mediated attenuation of Tat inhibitory effect by assessing zinc-regulation DA uptake and WIN35,428 binding, and functional basal substrate efflux. We found that D206L/H547A attenuated zinc-mediated increase in [^3^H]WIN35,428 binding and augmented basal efflux of substrate MPP+, whereas neither zinc-mediated increase in [^3^H]WIN35,428 binding nor basal MPP + efflux was altered in D206L. Moreover, our previous study shows that H547A attenuates both zinc-mediated [^3^H]DA uptake and [^3^H]WIN35,428 binding as well as increases basal substrate DA efflux (Quizon et al. 2016). Similarly, Y88F/D206L/H547A attenuated the zinc-mediated increase in [^3^H]WIN35,428 binding and had no effect on basal MPP + efflux, whereas Y88F did attenuate the zinc-mediated increase in [^3^H]WIN35,428 binding but had no effect on basal MPP + efflux (Midde et al. 2015), suggesting that the triple mutant may attenuate Tat effect through an independent mechanism. Consistent with the current results, our recent study has demonstrated that double mutations of Y88 and H547 (Y88F/H547A) attenuates the zinc-mediated [^3^H]DA uptake and [^3^H]WIN35,428 binding as well as increases basal substrate DA efflux (Sun et al. 2019). Thus, these findings significantly highlight the impact of H547A mutant on the allosteric modulation of Tat interaction with DAT. Next, we further characterized the role of H547A in transporter conformational transitions by assessing DA uptake with the accessibility to MTSET of an inserted cysteine (I159C) on a hDAT background. H547A with inserted MTSET-sensitive cysteine construct (E2C-I159C/H547A) increases the sensitivity of an inserted cysteine in the position 159 in DAT to MTSET-induced reduction in DA uptake compared to WT hDAT with the same inserted cysteine construct (E2C-I159C WT hDAT) or negative control (E2C-I159A/H547A). Our data indicate that DA translocation more readily occurs due to changing transporter conformation to outward-open status induced by the H547A mutation, which facilitates enhanced DAT uptake. Determining whether double mutant D206L/H547A induces transport conformational change by MTSET assay is an interesting topic for future investigations. On the other hand, we found that H547A displays enhanced the magnitude of 2-BP-induced decrease in DA uptake, suggesting that H547A-induced increased DA uptake is dependent on palmitoylation activity. This is consistent with our previous report showing H547A-induced alteration of basal PKC activity (Quizon et al. 2016).

In summary, our findings demonstrate that D206 in conjunction with His547 residues display a critical role in stabilizing basal DA transport and Tat-DAT interaction. The combination of these mutants retains single mutant H547A-mediated enhancement of DA transport and attenuates Tat inhibitory effect on DAT function. This study further demonstrates that an allosteric modulatory mechanism may contribute to the D206L/H547A-mediated attenuation of Tat effect. Thus, studying on the functional relevance of multiple mutation of the key DAT residues in Tat-DAT intermolecular interaction might open the possibilities for developing therapeutic compounds that block Tat binding to DAT with minimal influence on physiological DA transport in individuals with HAND.

## Supplementary Material

PMID: 33537927

## Figures and Tables

**Table 1:** Summary of kinetic properties and inhibitory activities in [^3^H]DA uptake in WT hDAT and mutants

	V_max_ (pmol/min/10^5^ cells)	K_m_ (μM)	IC_50_ (nM)		
			DA	Cocaine	GBR12909
WT hDAT	12.43±2.50	1.39±0.36	1810±490	350±60	300±110
D206L	13.09±3.55	1.66±0.78	1966±523	570±70	270±70
D381L	4.54±0.95*	1.04±0.34	338±38*	560±110	240±60

Data are presented as mean ± S.E.M. values from five to seven independent experiments performed in duplicate.

* *p* < 0.05, unpaired student’s *t* test compared to WT hDAT (n = 5–7)

**Table 2:** Summary of kinetic properties and inhibitory activities in[^3^PH]DA uptake in WT hDAT and mutants

	V_max_ (pmol/min/10^5^ cells)	K_m_ (μM)	IC_50_ (nM)		
			DA	Cocaine	GBR12909
WT hDAT	26.62±3.87	0.45±0.21	467±85	152±48	660±84
D206L/H547A	40.82±3.60*	0.69±0.10	695±37	141±67	477±85
Y88F/D206L/H547A	10.78±0.98*	1.92±0.3*	1830±58*	121±30	180±18*

Data are presented as mean ± S.E.M. values from ten independent experiments performed in duplicate.

* *p* < 0.05, unpaired student’s *t* test compared to WT hDAT

**Table 3:** Summary of kinetic properties and inhibitory activities in [^3^H]WIN35,428 binding in WT hDAT and mutants

	B_max_ (pmol/10P^5^P cells)	K_d_ (μM)	IC_50_ (nM)		
			DA	Cocaine	GBR12909
WT hDAT	3.23±0.58	5.98±0.73	1350±382	308±55	770±193
D206L	2.87±0.96	6.37±1.42	1882±409	566±116	1618±456
D381L	2.00±0.63	17.52±6.57*	670±292	1480±276***	2620±404**

Data are presented as mean ± S.E.M. values from five to seven independent experiments performed in duplicate.

* *p* < 0.05,

** *p* < 0.01,

*** *p* < 0.001 unpaired student’s *t* test compared to WT hDAT (n = 6–12)

**Table 4:** Summary of kinetic properties in [^3^H]WIN35,428 binding in WT hDAT and mutants

	B_max_ (poml/10^5^ cells)	K_d_ (μM)
WT hDAT	2.44±0.40	12.53±1.88
D206L/H547A	4.95±1.28	10.95±0.91
Y88F/D206L/H547A	3.50±0.47	6.90±1.26*

Data are presented as mean ± S.E.M. values from five to seven independent experiments performed in duplicate.

* *p* < 0.05, unpaired student’s *t* test compared to WT hDAT (n = 6–12)

**Table 5:** Inhibitory effects of palmitoylation activity on the kinetic parameters of [^3^H]DA uptake in WT hDAT and H547A

	V_max_ (pmol/min/10^5^ cells)			
	WT hDAT		H547A	
	Veh	2-BP	Veh	2-BP
0 h	4.14±0.57	5.36±0.74	6.55±1.19	6.41±1.45
2 h	8.80±0.92	5.55±0.92*	16.33±0.99^#^	5.66±1.93*
	K_m_ (μM)			
	WT hDAT		H547A	
	Veh	2-BP	Veh	2-BP
0 h	0.53±0.10	0.66±0.12	0.88±0.12	0.81±0.08
2 h	0.76±0.07	0.66±0.12	1.84±0.19^#^	2.31±0.84

Data are presented as mean ± S.E.M. values from three to four independent experiments performed in duplicate.

* *p* < 0.05, compared to the respective vehicle control (Veh).

# *p* < 0.05, compared to WT HDAT within vehicle group
