# Supplementary material for: Mutations of Human DopamineTransporter at Tyrosine88, Aspartic Acid206, and Histidine547 Influence Basal and HIV-1 Tat‐inhibited Dopamine Transport
Source: J Neuroimmune Pharmacol. 2021 Feb 3;16(4):854–69. doi: 10.1007/s11481-021-09984-5 (PMC8329121; doi:10.1007/s11481-021-09984-5)
Supplement: Supplementary file 1 — (DOCX 3.29 MB) [file 11481_2021_9984_MOESM1_ESM.docx]

**Mutations of human dopamine transporter at tyrosine88,** **aspartic acid206, and histidine547 influence basal and HIV-1 Tat-inhibited dopamine transport**

Pamela M. Quizon1, Yaxia YuanP^2,3^P, Yike ZhuP^1^P, Yi ZhouP^1^P, Matthew J. StraussP^1^P, Wei-Lun SunP^1^P, Chang-Guo ZhanP^2,3^, and Jun ZhuP^1*^

P^a^Department of Drug Discovery and Biomedical Sciences, College of Pharmacy, University of South Carolina, Columbia, SC.

P^b^Molecular Modeling and Biopharmaceutical Center, and ^c^Department of Pharmaceutical Sciences, College of Pharmacy, University of Kentucky, Lexington, KY

*Corresponding Author:

Jun Zhu, MD., PhD

Department of Drug Discovery and Biomedical Sciences

College of Pharmacy

University of South Carolina

715 Sumter Street, Columbia, SC 29208, USA.

Tel: +1-803-777-7924; Fax: +1-803-777-8356

E-mail: [zhuj@sccp.sc.edu](mailto:zhuj@sccp.sc.edu)


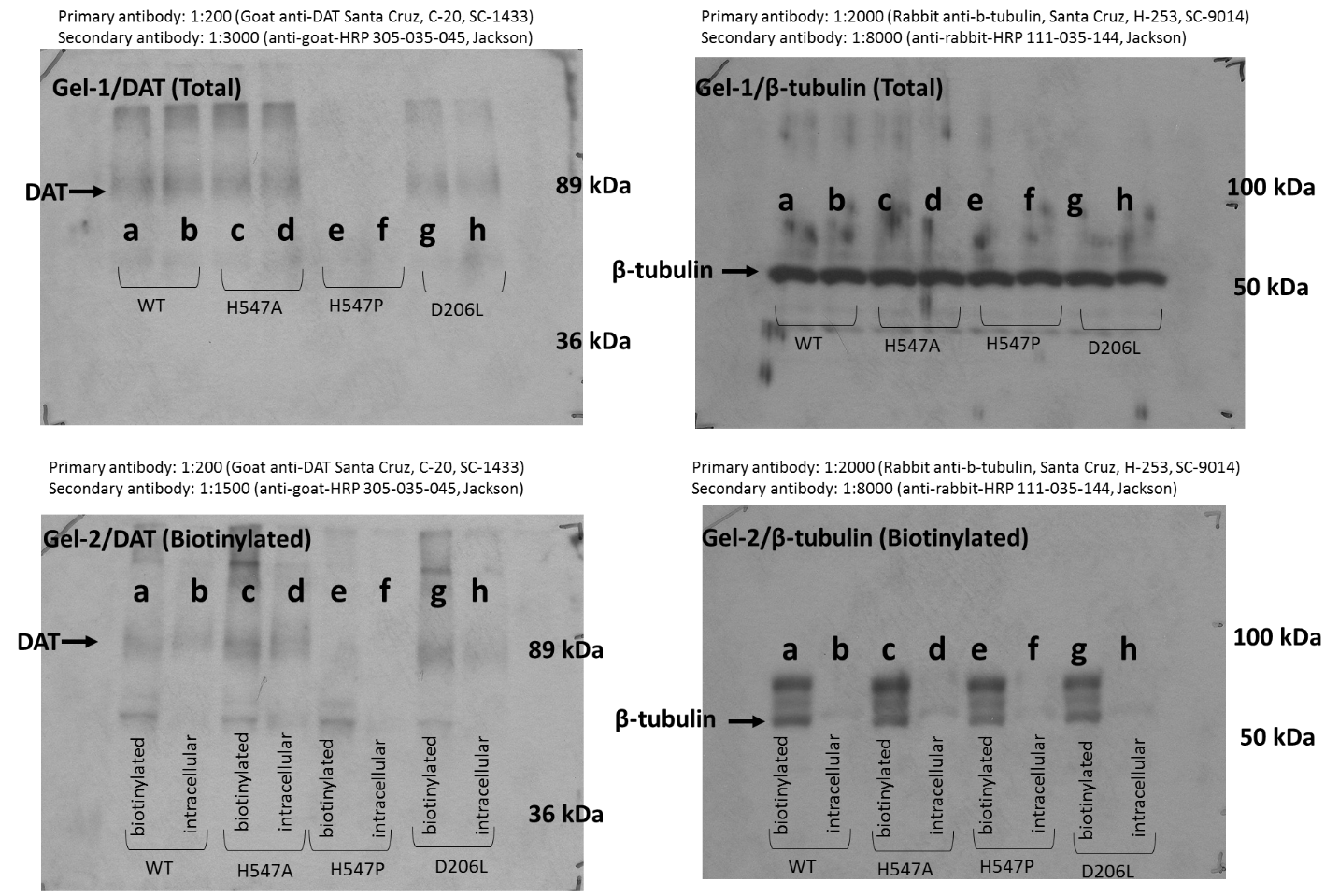


**Supplementary Figure 1**. Representative immunoblots of total and biotinylated WT hDAT and D206L.

Top-left: Total DAT expression in PC12 cells expressing WT hDAT or mutant. The total fraction was loaded in duplicate onto the gel under the same experimental conditions. Lanes **a** and **g** were cropped to represent Total DAT for WT and D206L, respectively, in **Figure 4A (left)**.

Bottom-left: Biotinylated (surface) and intracellular DAT expression in PC12 cells expressing WT hDAT or mutant. The biotinylated and intracellular fractions were loaded in duplicate onto the gel under the same experimental conditions. Lanes **a** and **g** were cropped to represent Biotinylated DAT for WT and D206L, respectively, in **Figure 4A (left)**.

Blots on right: β-tubulin antibody was used to re-probe the corresponding blots on the left in order to show that equal amounts of protein were loaded on each sample lane.


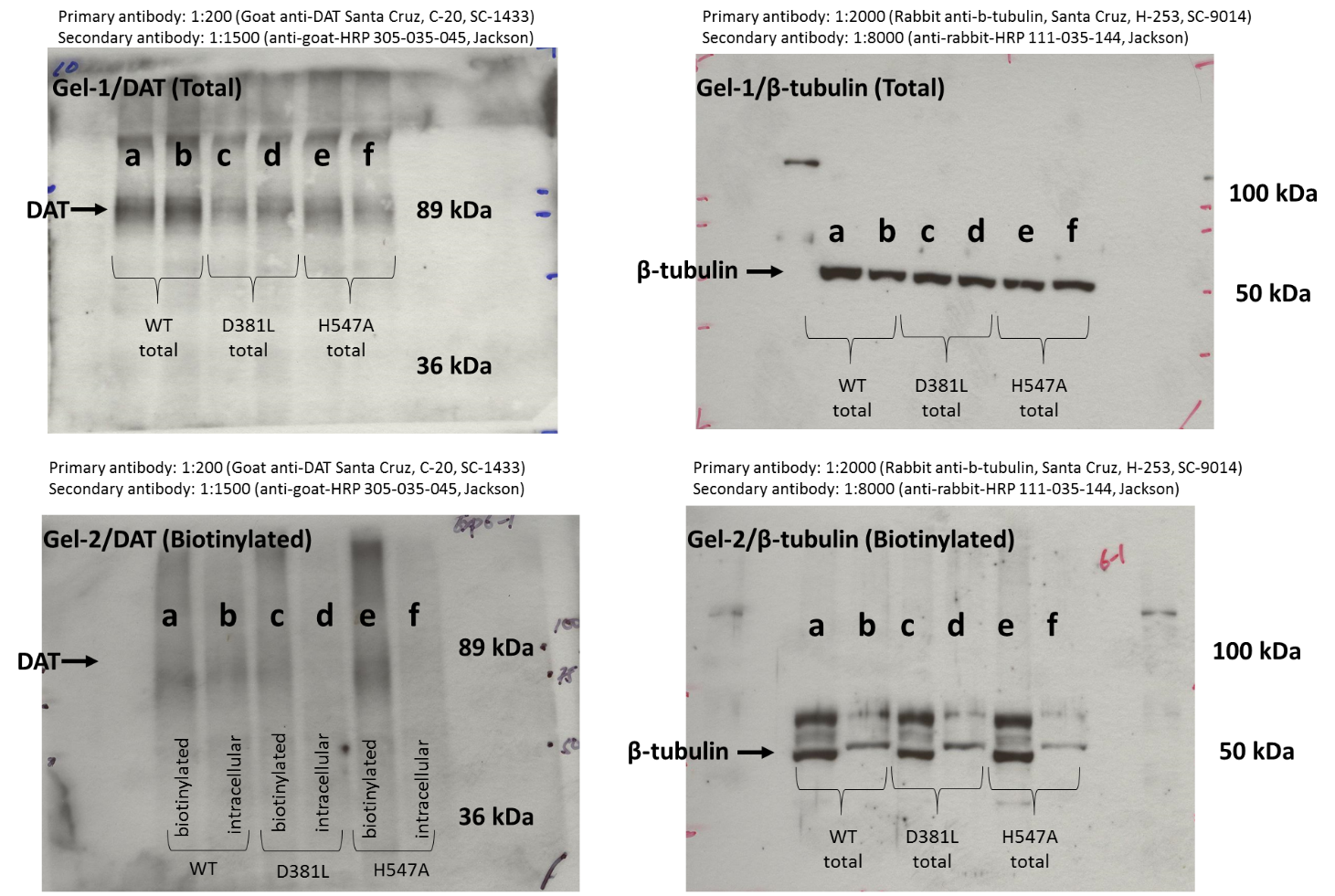


**Supplementary Figure 2**. **Representative immunoblots of total and biotinylated WT hDAT (a, b) and D381L (c, d).**

Top-left: Total DAT expression in PC12 cells expressing WT hDAT or mutant. The total fraction was loaded in duplicate onto the gel under the same experimental conditions. Lanes **a** and **c** were cropped to represent Total DAT for WT and D381L, respectively, in **Figure 4A (right)**.

Bottom-left: Biotinylated (surface) and intracellular DAT expression in PC12 cells expressing WT hDAT or mutant. The biotinylated and intracellular fractions were loaded in duplicate onto the gel under the same experimental conditions. Lanes **a** and **c** were cropped to represent Biotinylated DAT for WT and D381L, respectively, in **Figure 4A (right)**.

Blots on right: β-tubulin antibody was used to re-probe the corresponding blots on the left in order to show that equal amounts of protein were loaded on each sample lane.


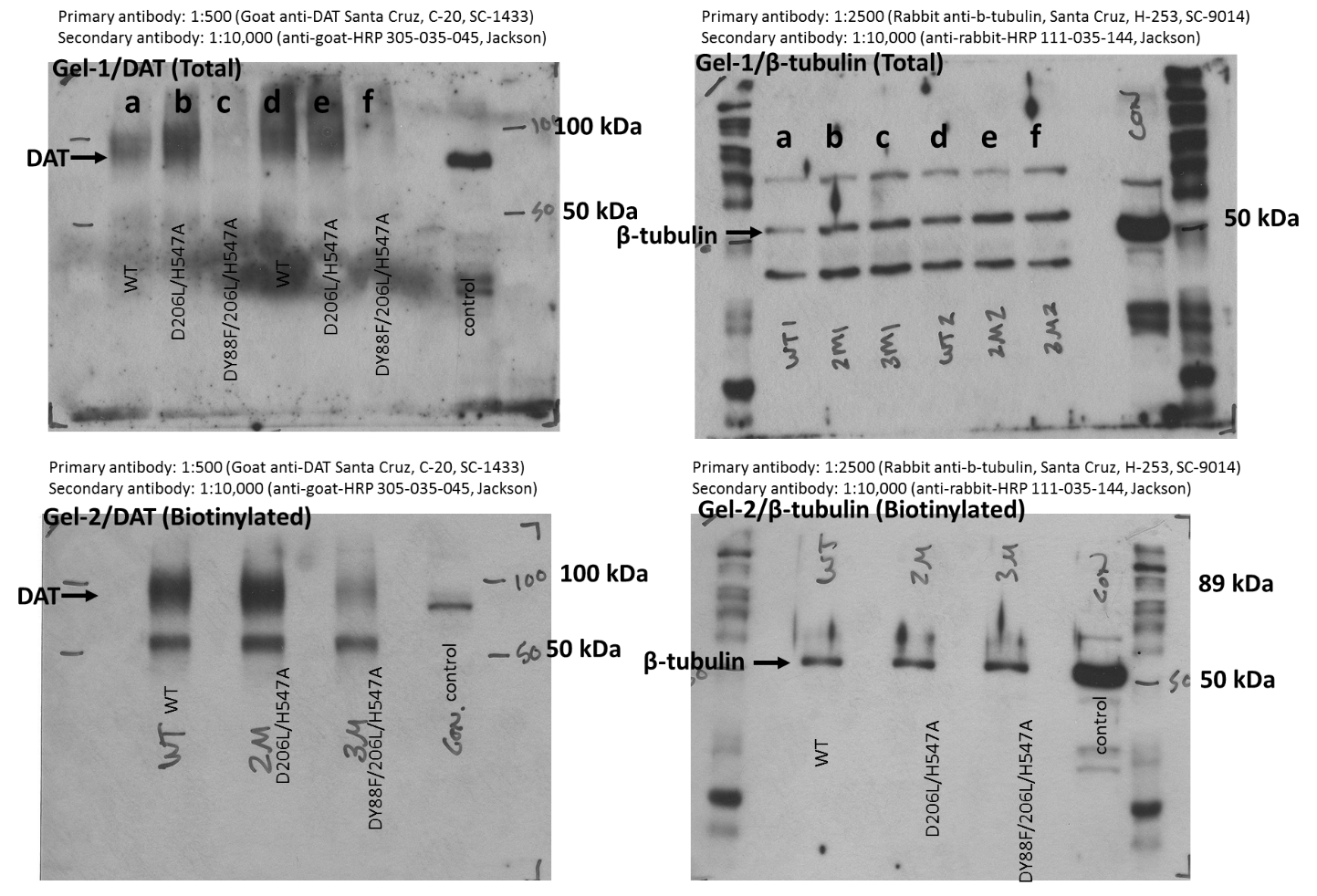


**Supplementary Figure 3**. **Representative immunoblots of total and biotinylated WT hDAT, D206L/H547A, and Y88/D206L/H547A.**

Top-left: Total DAT expression in PC12 cells expressing WT hDAT or mutant. The total fraction was loaded in duplicate onto the gel under the same experimental conditions. Lanes **d**, **e**, and **f** were cropped to represent Total DAT for WT, D206L/H547A, and Y88F/D206L/H547A, respectively, in **Figure 4B**.

Bottom-left: Biotinylated (surface) and intracellular DAT expression in PC12 cells expressing WT hDAT or mutant. The biotinylated and intracellular fractions were loaded in duplicate onto the gel under the same experimental conditions. The WT, D206L/H547A, and Y88F/D206L/H547A lanes were cropped to represent Biotinylated DAT in **Figure 4B**.

Blots on right: β-tubulin antibody was used to re-probe the corresponding blots on the left in order to show that equal amounts of protein were loaded on each sample lane.
