# Supplementary material for: Mutations of Human Dopamine Transporter at Tyrosine88, Aspartic Acid206, and Histidine547 Influence Basal and HIV-1 Tat-inhibited Dopamine Transport
Source: J Neuroimmune Pharmacol. Author manuscript; Available in PMC 2021 Dec 29. (PMC8329121; doi:10.1007/s11481-021-09984-5)
Supplement: Supplementary file 1 [file NIHMS1684684-supplement-PMID__33537927.pdf]

Mutations of human dopamine transporter at tyrosine88, aspartic acid206, and histidine547 influence basal and HIV-1 Tat-inhibited dopamine transport

Pamela M. Quizon, Yaxia Yuan[2,3], Yike Zhu[1], Yi Zhou[1], Matthew J. Strauss[1], Wei-Lun Sun[1], Chang-Guo Zhan[2,3], and Jun Zhu[1*]

[a]Department of Drug Discovery and Biomedical Sciences, College of Pharmacy, University of South Carolina, Columbia, SC.
[b]Molecular Modeling and Biopharmaceutical Center, and [c]Department of Pharmaceutical Sciences, College of Pharmacy, University of Kentucky, Lexington, KY

*Corresponding Author:
Jun Zhu, MD., PhD
Department of Drug Discovery and Biomedical Sciences
College of Pharmacy
University of South Carolina
715 Sumter Street, Columbia, SC 29208, USA.
Tel: +1-803-777-7924; Fax: +1-803-777-8356
E-mail: zhuj@sccp.sc.edu

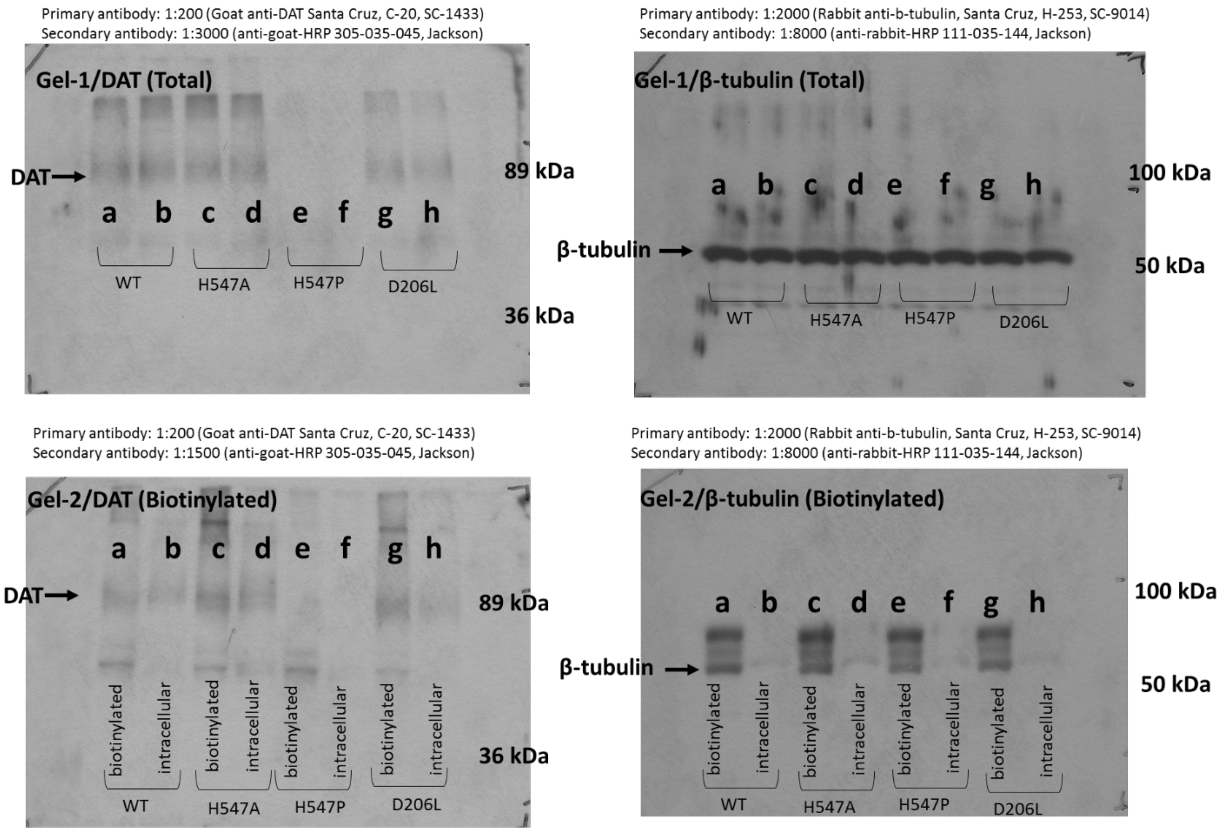

Supplementary Figure 1. Representative immunoblots of total and biotinylated WT hDAT and D206L. Top-left: Total DAT expression in PC12 cells expressing WT hDAT or mutant. The total fraction was loaded in duplicate onto the gel under the same experimental conditions. Lanes **a** and **g** were cropped to represent Total DAT for WT and D206L, respectively, in **Figure 4A (left)**.
Bottom-left: Biotinylated (surface) and intracellular DAT expression in PC12 cells expressing WT hDAT or mutant. The biotinylated and intracellular fractions were loaded in duplicate onto the gel under the same experimental conditions. Lanes **a** and **g** were cropped to represent Biotinylated DAT for WT and D206L, respectively, in **Figure 4A (left)**.
Blots on right: β-tubulin antibody was used to re-probe the corresponding blots on the left in order to show that equal amounts of protein were loaded on each sample lane.

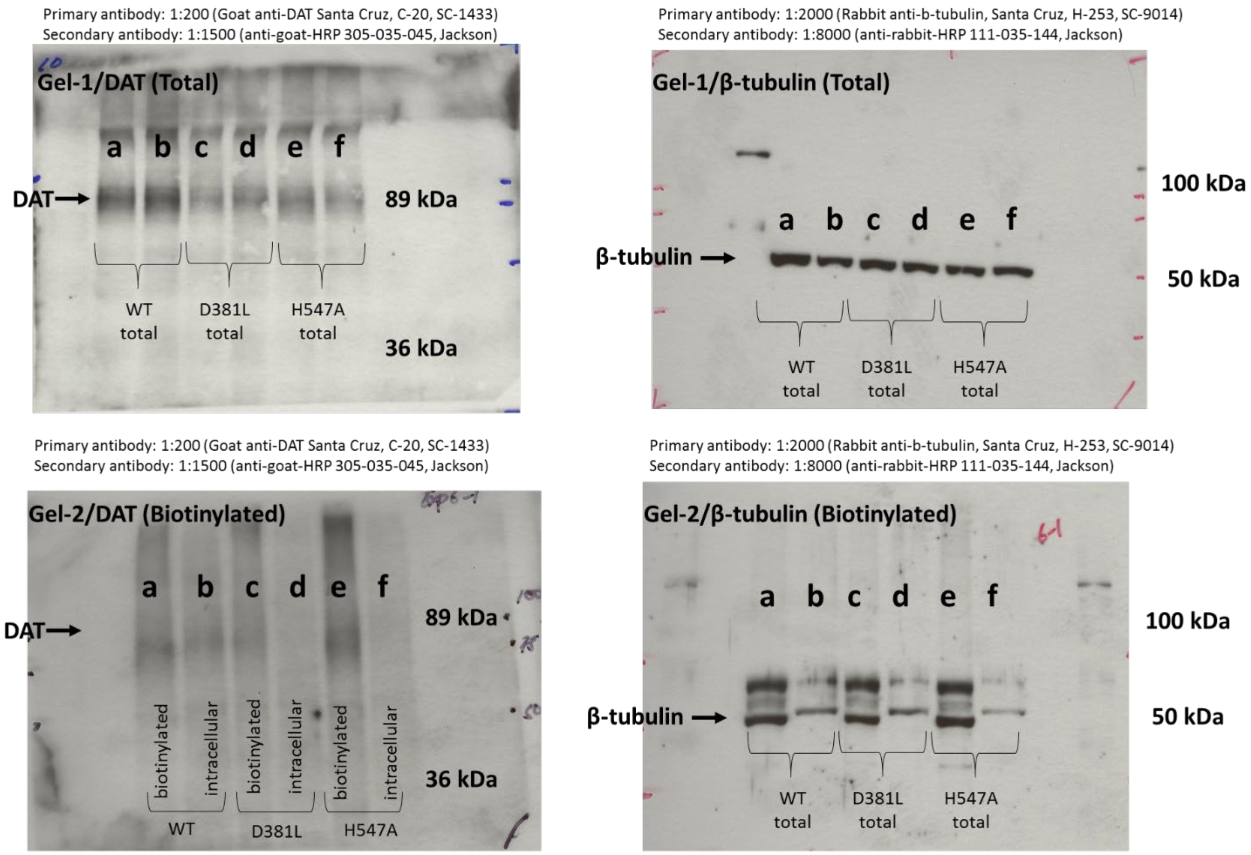

Supplementary Figure 2. **Representative immunoblots of total and biotinylated WT hDAT (a, b) and D381L (c, d).**
Top-left: Total DAT expression in PC12 cells expressing WT hDAT or mutant. The total fraction was loaded in duplicate onto the gel under the same experimental conditions. Lanes **a** and **c** were cropped to represent Total DAT for WT and D381L, respectively, in **Figure 4A (right)**.
Bottom-left: Biotinylated (surface) and intracellular DAT expression in PC12 cells expressing WT hDAT or mutant. The biotinylated and intracellular fractions were loaded in duplicate onto the gel under the same experimental conditions. Lanes **a** and **c** were cropped to represent Biotinylated DAT for WT and D381L, respectively, in **Figure 4A (right)**.
Blots on right: β-tubulin antibody was used to re-probe the corresponding blots on the left in order to show that equal amounts of protein were loaded on each sample lane.

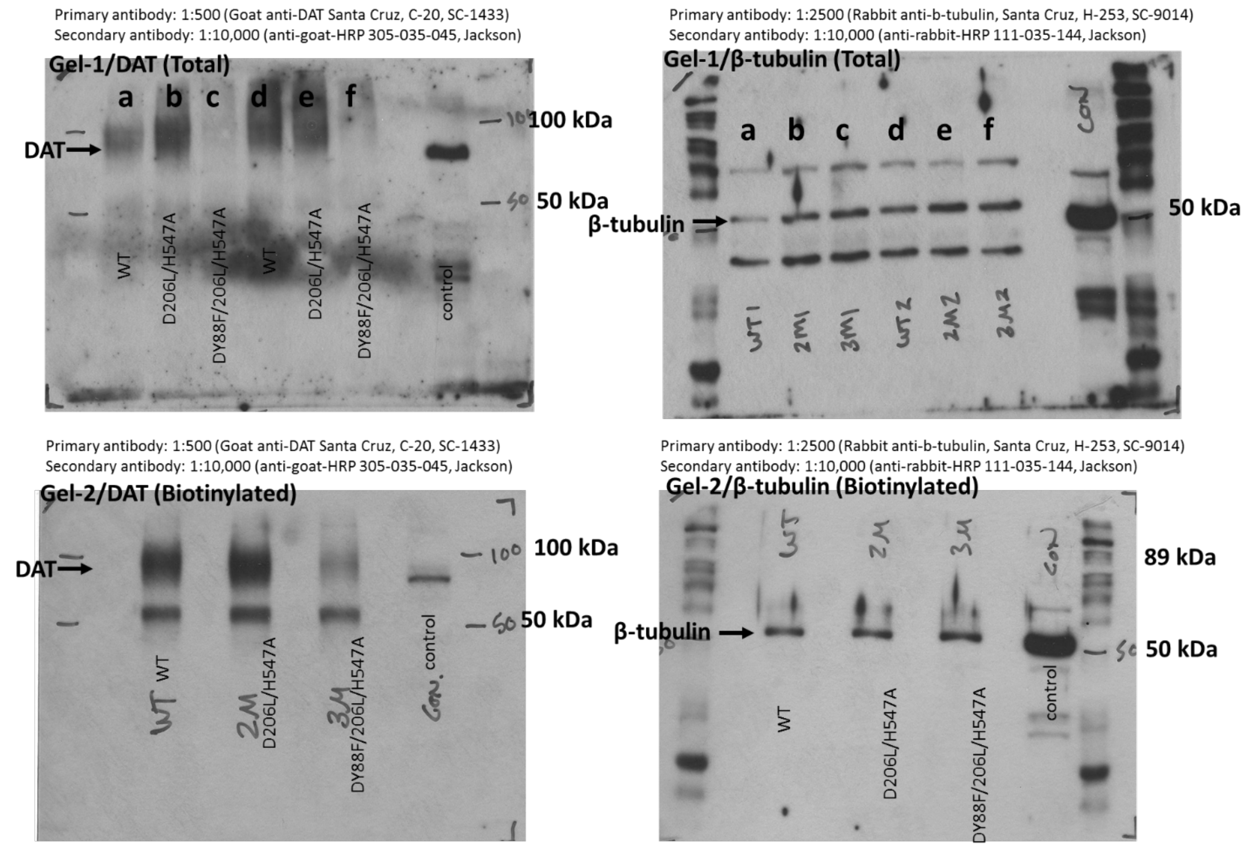

Supplementary Figure 3. **Representative immunoblots of total and biotinylated WT hDAT, D206L/H547A, and Y88/D206L/H547A.**

Top-left: Total DAT expression in PC12 cells expressing WT hDAT or mutant. The total fraction was loaded in duplicate onto the gel under the same experimental conditions. Lanes **d**, **e**, and **f** were cropped to represent Total DAT for WT, D206L/H547A, and Y88F/D206L/H547A, respectively, in **Figure 4B**.

Bottom-left: Biotinylated (surface) and intracellular DAT expression in PC12 cells expressing WT hDAT or mutant. The biotinylated and intracellular fractions were loaded in duplicate onto the gel under the same experimental conditions. The WT, D206L/H547A, and Y88F/D206L/H547A lanes were cropped to represent Biotinylated DAT in **Figure 4B**.

Blots on right: β-tubulin antibody was used to re-probe the corresponding blots on the left in order to show that equal amounts of protein were loaded on each sample lane.
